# Tomato *SlBL4* plays an important role in fruit pedicel organogenesis and abscission

**DOI:** 10.1038/s41438-021-00515-0

**Published:** 2021-04-01

**Authors:** Fang Yan, Zhehao Gong, Guojian Hu, Xuesong Ma, Runyao Bai, Ruonan Yu, Qiang Zhang, Wei Deng, Zhengguo Li, Hada Wuriyanghan

**Affiliations:** 1 Key Laboratory of Herbage & Endemic Crop Biotechnology, Ministry of Education, School of Life Science, Inner Mongolia University, Hohhot, 010021 China; 2 Key Laboratory of Plant Hormones and Development Regulation of Chongqing, School of Life Sciences, Chongqing University, 401331 Chongqing, China; 3 School of Biology and Basic Medical Sciences, Soochow University, Suzhou, China; 4 Center of Plant Functional Genomics, Institute of Advanced Interdisciplinary Studies, Chongqing University, 401331 Chongqing, China

**Keywords:** RNAi, Plant molecular biology

## Abstract

Abscission, a cell separation process, is an important trait that influences grain and fruit yield. We previously reported that *BEL1-LIKE HOMEODOMAIN 4* (*SlBL4*) is involved in chloroplast development and cell wall metabolism in tomato fruit. In the present study, we showed that silencing *SlBL4* resulted in the enlargement and pre-abscission of the tomato (*Solanum lycopersicum cv. Micro-TOM*) fruit pedicel. The anatomic analysis showed the presence of more epidermal cell layers and no obvious abscission zone (AZ) in the *SlBL4* RNAi lines compared with the wild-type plants. RNA-seq analysis indicated that the regulation of abscission by *SlBL4* was associated with the altered abundance of genes related to key meristems, auxin transporters, signaling components, and cell wall metabolism. Furthermore, *SlBL4* positively affected the auxin concentration in the abscission zone. A dual-luciferase reporter assay revealed that SlBL4 activated the transcription of the *JOINTLESS*, *OVATE*, *PIN1,* and *LAX3* genes. We reported a novel function of SlBL4, which plays key roles in fruit pedicel organogenesis and abscission in tomatoes.

## Introduction

Organ abscission is critical for plant growth and development, as it enables the recycling of nutrients for continuous growth, development of appropriate organs, survival in case of disease, and reproduction^1,2^. Plant organ shedding refers to the abscission of some plant organs, such as flowers, leaves, fruits, and other tissues; it is caused by the coordinated actions of physiological processes, biochemical metabolism, and gene regulatory networks^3^. Abscission occurs at predetermined positions called abscission zones (AZs), and the abscission process includes differentiation of the AZ, acquisition of the competence to respond to abscission signals, activation of organ abscission, and formation of a protective layer^4–7^. Abscission initiation is considered to be triggered by the interaction of two hormones, auxin and ethylene^8–10^. During the late abscission stages, several key enzymes play an important role in organ shedding. Cellulase (Cel) and polygalacturonase (PG) participate in the degradation of the cell wall, and pectin methylesterase (PME) changes the chemical structure of the AZ via hydrolysis and induces cell wall and membrane degradation^11^.

Genetic analyses of tomato (*Solanum lycopersicum*) have revealed that many transcription factors (TFs) are involved in AZ differentiation and abscission. *JOINTLESS* was shown to be directly related to the development of flower pedicels, inflorescence structure, fruit shape, and seed development, and the *jointless* mutant failed to develop AZs^12,13^. In addition to *JOINTLESS*, *jointless-2* delayed the development and formation of tomato AZs^13^. Two MADS-box genes, *Macrocalyx* (*MC*) and *SlMBP21*, regulate pedicel AZ development, and the knockdown of these genes results in a *jointless* phenotype^14,15^. *BLIND* (*Bl*), a R2R3-class MYB TF gene, genetically interacts with *JOINTLESS* and plays an important role in abscission^14,16,17^. *Lateral suppressor* (*Ls*) partially impairs AZ development and causes malformation of meristem axillary buds. Furthermore, tomato *ls* mutants lack petals in their flowers^18^. *BLADE ON PETIOLE1/2* (*BOP1* and *BOP2*) were shown to be involved in the formation of *Arabidopsis thaliana* floral organ AZs, and the floral organs failed to abscise in the *bop1/bop2* double mutant^19^.

The three-amino-acid-loop-extension (TALE) class genes encode TFs, such as KNOTTED-like (KNOX) and BEL1-like (BLH, BELL), and are typically involved in the regulation of meristematic activity^20^. TALE homeobox genes not only mediate plant development but also participate in plant organ separation. In *A. thaliana*, several members of the TALE family are reported to play central roles in regulating pedicel development. The *KNAT*/*BP* gene affects the development of *A. thaliana* floral AZs. In the *bp* mutant, the floral organs form more follicular cells and are abscised early due to the increased expression of *KNAT2* and *KNAT6* in the pedicel^21–23^. *A. thaliana homeobox* gene 1 (*ATH1*), a BELL TF member, plays a key role in the KNAT2 pathway to regulate pedicel development^24^. PENNYWISE (PNY) and POUND-FOOLISH (PNF) form heterodimers with KNOX proteins to regulate flowering initiation and inflorescence architecture^25–28^. *SlBL4*, a tomato bell-like gene, was shown to target chlorophyll synthesis and cell wall metabolism genes to control chloroplast development and cell wall metabolism in tomato fruit^29^. The expression of *SlBL4* was upregulated in AZs^10^, and SlBL4 has high sequence similarity with ATH1^29^, suggesting that it plays an important role in tomato pedicel AZ development.

Tomato is an excellent model for the study of the AZ, as it has distinct fruit/flower pedicels, rich genetic resources, and a stable genetic background. The present work identified a previously undefined role of the tomato BELL family gene *bell-like homeodomain protein 4* (*SlBL4*) in the development of fruit pedicels. The pedicel AZ expanded more after anthesis, and the rate of fruit abscission was significantly increased starting from the abscission day in *SlBL4* RNAi plants compared with WT plants. AZ transcriptomic and physiological analyses showed that the SlBL4 protein might play a role in the initiation and abscission of tomato AZs by regulating a variety of gene families and cell wall substructures.

## Materials and methods

### Plant material and growth conditions

*Micro-Tom* tomato plants (*Solanum lycopersicum*) were grown under greenhouse conditions with a 16 h light (25 °C ± 2 °C)/8 h dark (18 °C ± 2 °C) cycle and 80% relative humidity with 250 μmol m^–2^ s^–1^ of intense light.

### Quantitative RT-PCR (qRT-PCR) analysis, plant binary vector construction, and tomato transformation

For the expression analysis of *SlBL4* in tomato pedicels at different stages, the materials were collected, immediately frozen in liquid nitrogen, and stored at −80 °C for RNA extraction. Gene-specific primers are listed in Table S1. qRT-PCR was carried out as described previously^30^.

The promoter sequence of *SlBL4* was amplified from tomato genomic DNA with the primers *SlBL4-PF* and *SlBL4-PR* (Table S1). The promoter fragment of *SlBL4* was digested with *Sal* I/*Bam*H I and ligated into the plant binary vector pLP100 containing the *GUS* reporter gene, yielding the reporter vector pLP100p*SlBL4-GUS*. Transgenic plants were obtained by the *Agrobacterium*-mediated transformation method^31^. The transgenic lines were selected and confirmed by qPCR and GUS staining according to the methods of Yan^32^. The *SlBL4*-RNAi plants were obtained according to the method of a previous study^29^. Three representative transgenic lines (L19, L22, and L23) were selected for further analysis, and all experiments were performed using homozygous lines of the T_3_ generation.

### Auxin and ethylene treatment

For auxin treatment, tomato seeds (*n* = 30) from the wild type (WT) and *SlBL4* RNAi lines were soaked in MS medium supplemented with different concentrations (0, 0.25, 0.5, and 1 μM) of indole-3-acetic acid (IAA) (Sigma, USA) for 14 days in a culture chamber. Primary and lateral root numbers and lengths were measured, and photographs were taken after 14 days of growth. For ethylene treatment, the same seeds were soaked in MS medium supplemented with 1 μM ACC for 7 days in the dark culture chamber as described above. Root and hypocotyl elongation were observed and measured after 7 days of growth. All experiments were independently repeated at least three times.

Floral explants were prepared by excising freshly opened flowers, including the pedicel AZ. For the IAA treatment, the pedicel ends of explants were inserted into a layer of 1/2 MS agar with 50 μg/g IAA in a Plexiglass box and placed in a tray filled with a layer of water. For the ethylene treatment, the pedicel ends of explants were treated similarly, but ethylene gas was added to the box at a final concentration of 20 µl/L. The abscised pedicel explants were counted at 8, 16, 24, 32, 40, 48, 56, 64, 72, 84, 96, 108, and 120 h after treatment. Three biological replicates were performed, and each treatment group contained ~50 explants.

### GUS Staining and auxin content measurement

Tomato inflorescences were placed into GUS staining buffer comprising 2.0 mM 5-bromo-4-chloro-3-indolyl-b-glucuronic acid, 0.1 M Na_3_PO_4_ (pH 7.0), 1.0 mM K_3_ Fe (CN)_6_, 10 mM Na_2_EDTA and 0.1% (v/v) Triton X-100. The inflorescences were vacuumed for 30 min and incubated in the dark at 37 °C for 16 h. GUS-stained tissues were washed with 70% (v/v) ethanol and observed under a light microscope.

Floral explants including the pedicel AZ were prepared by excising the tissues at 6, 4, and 2 days before anthesis (dba), on the anthesis day, and at 2 and 25 days post anthesis (dpa). For IAA treatment, the pedicel ends of explants were inserted into a layer of 1/2 MS agar with 50 μg/g IAA, and the floral explants were stained with GUS after 24 h of treatment.

The IAA content was measured by acquisition ultraperformance liquid chromatography (Acquity UPLC; Waters). Fifty pedicel AZ segments were collected from the pedicel at the abscission stage, immediately frozen in liquid nitrogen, and stored at −80 °C for IAA quantification according to the literature^33^.

### Microscopy and TEM observation

For histological analysis, tissue samples were collected from the pedicel at 25 dpa and immersed in FAA solution, placed under vacuum for 15 min, and incubated at 25 °C for 72 h. The samples were dehydrated, stained, and observed under a microscope according to the literature^32^. At anthesis, flowers of WT plants and *SlBL4* RNAi line tomatoes were emasculated and subsequently counted and sampled for anatomical assessment at 10 dpa. For the histological assessment of *SlBL4*pro::*GUS* expression, tissue samples were collected from the pedicel of p*SlBL4*-*GUS* transgenic tomato at the following times: at 6, 4, 2 dba; on the anthesis day; and at 2, 25 dpa. The pedicel of the AZ at 25 dpa was observed by transmission electron microscopy (TEM) according to the literature^34^.

### RNA-seq analysis

Pedicel samples, including those at three stages (0 dpa, 25 dpa, and the fruit break stage), were dissected using a sharp razor blade into three segments (distal, AZ, and proximal tissues), and an AZ segment of ~2 mm was placed in liquid nitrogen immediately. Total RNA was extracted using the RNeasy Plant Mini Kit (Qiagen, USA) according to the manufacturer’s instructions. Three biological replicates were performed for each sample for both *SlBL4*-RNAi and WT plants. The RNA-Seq libraries were constructed and sequenced on the Illumina HiSeq 2000 platform at the Wuhan Genome Institute (BGI, China). The raw sequences were processed by removing the adaptor and low-quality sequences. The expression levels of DEGs were normalized by the fragments per kilobase of exon per million mapped reads (FRKM) method using Cuffdiff software (http://cole-trapnell-lab.github.io/cufflinks/). A false discovery rate (FDR) ≤ 0.05 was used to determine the threshold for DEGs. GO functional enrichment and KEGG pathway analyses were conducted according to previously described methods (https://github.com/tanghaibao/goatools) and KOBAS (http://kobas.cbi.pku.edu.cn/home.do).

### Dual-luciferase transient expression assay

For effector vector construction, the full-length coding sequence of *SlBL4* was amplified and then cloned into the pEAQ-Empty vector^29^. For reporter vector construction, the promoters of the *JOINTLESS*, *OVATE*, *LAX3*, and *PIN1* genes were cloned into a pGreenII 0800-LU vector^35^. A dual-luciferase transient expression assay for SlBL4 was carried out using tobacco leaves (*Nicotiana benthamiana*). A dual-luciferase assay kit (Promega, USA) was used to measure the activities of LUC and REN luciferase according to the manufacturer’s instructions on a Luminoskan Ascent microplate luminometer (Thermo Fisher Scientific, USA). Six biological repeats were performed for each pair of vector combinations. The primer sequences used for the vector construct are shown in Table S1.

## Results

### *SlBL4* expression is associated with AZ development

**Fig. 1:**
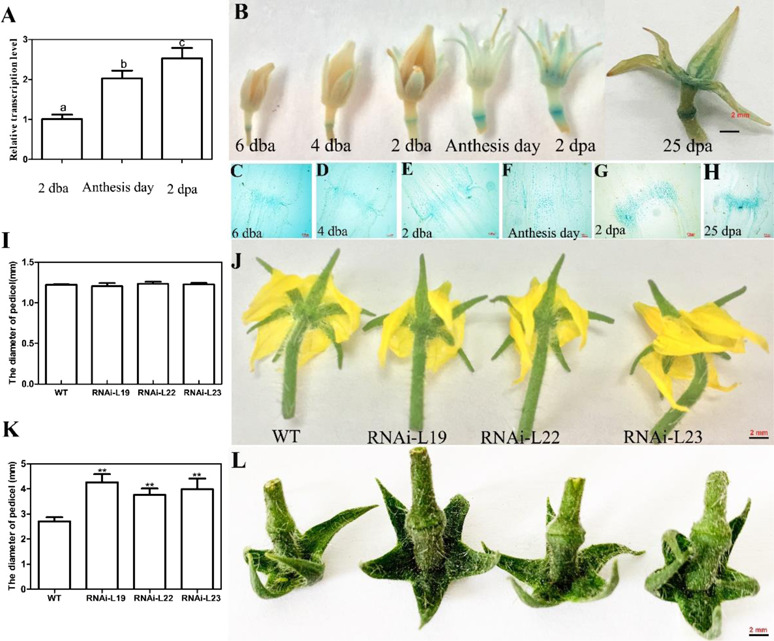
Expression analysis of *SlBL4* and the pedicel abscission phenotype of the RNAi plants. **A** Expression analysis of *SlBL4* in different stages of tomato pedicel abscission; 2 dba, 2 days before anthesis; 2 dpa, 2 days post anthesis. The quantitative PCR data represent mean values for three independent biological replicates, and Duncan’s multiple range test was performed to compare samples in different groups. The standard errors are indicated by vertical bars. **B** *SlBL4*pro::*GUS* expression at 6, 4, 2 dba: 6, 4, and 2 days before anthesis; anthesis day; 2, 25 dpa: 2 and 25 days post anthesis; **C**–**H** Anatomic analysis of the floral pedicel abscission zone of *SlBL4*pro::*GUS* plants: **C** 6 dba; **D** 4 dba; I 2 dba; **F** anthesis day; **G** 2 dpa; **H** 25 dpa. **I** The diameter of the pedicel abscission zone on the anthesis day. **J** Anthesis day pedicel. **K** The diameter of the pedicle abscission zone on 25 dpa. **L** 25 dpa pedicel. WT wild type, RNAi-L19, RNAi-L22, RNAi-L23 three different lines of *SlBL4* RNAi plants. Scale bars: 2 mm

*SlBL4* was reported to be predominately expressed in pedicel AZs in tomato^10^. To clarify the involvement of *SlBL4* in tomato pedicel abscission, the transcript level of *SlBL4* was analyzed in pedicel AZs from the floral bud stage to the flowering stage (2 days before anthesis, 2 dba; anthesis day, AS; 2 days post anthesis, 2 dpa) by qRT-PCR. The transcript levels of *SlBL4* showed progressive increases at 2 dba, AS and 2 dpa (Fig. 1A). Furthermore, p*SlBL4-GUS* transgenic tomato was generated according to the literature^32^. Consistent with the qRT-PCR results, *GUS* expression was obviously visible in the AZ and showed an increasing expression pattern during anthesis development (Fig. 1B). The anatomic analysis was performed to more precisely assess the *SlBL4* expression pattern in the AZ. The results showed that *GUS* expression was obviously visible in the whole pedicle before anthesis and increased after anthesis in the pedicle AZ (Fig. 1C–H). This AZ-specific expression pattern indicated that *SlBL4* possibly participated in the abscission process.

### Silencing *SlBL4* augmented the pedicel and promoted abscission

In this study, we used a *35S*::*SlBL4*-RNAi silencing construct to generate *SlBL4* knockdown *Micro-Tom* tomato plants. The transgenic tomato exhibited *SlBL4* expression that was reduced by 20-80% compared with that of the WT plant^29^. At 25 days after flowering, the floral pedicel AZ expanded to a greater extent in *SlBL4* knockdown plants than in the control WT plants, whereas no obvious difference was found at the anthesis stage (Fig. 1C–F). The AZ diameters were 3.76–4.26 mm in the three *SlBL4* RNAi lines and 39–57% higher than those in the WT plants at 25 days after flowering (Fig. 1E, F).

**Fig. 2:**
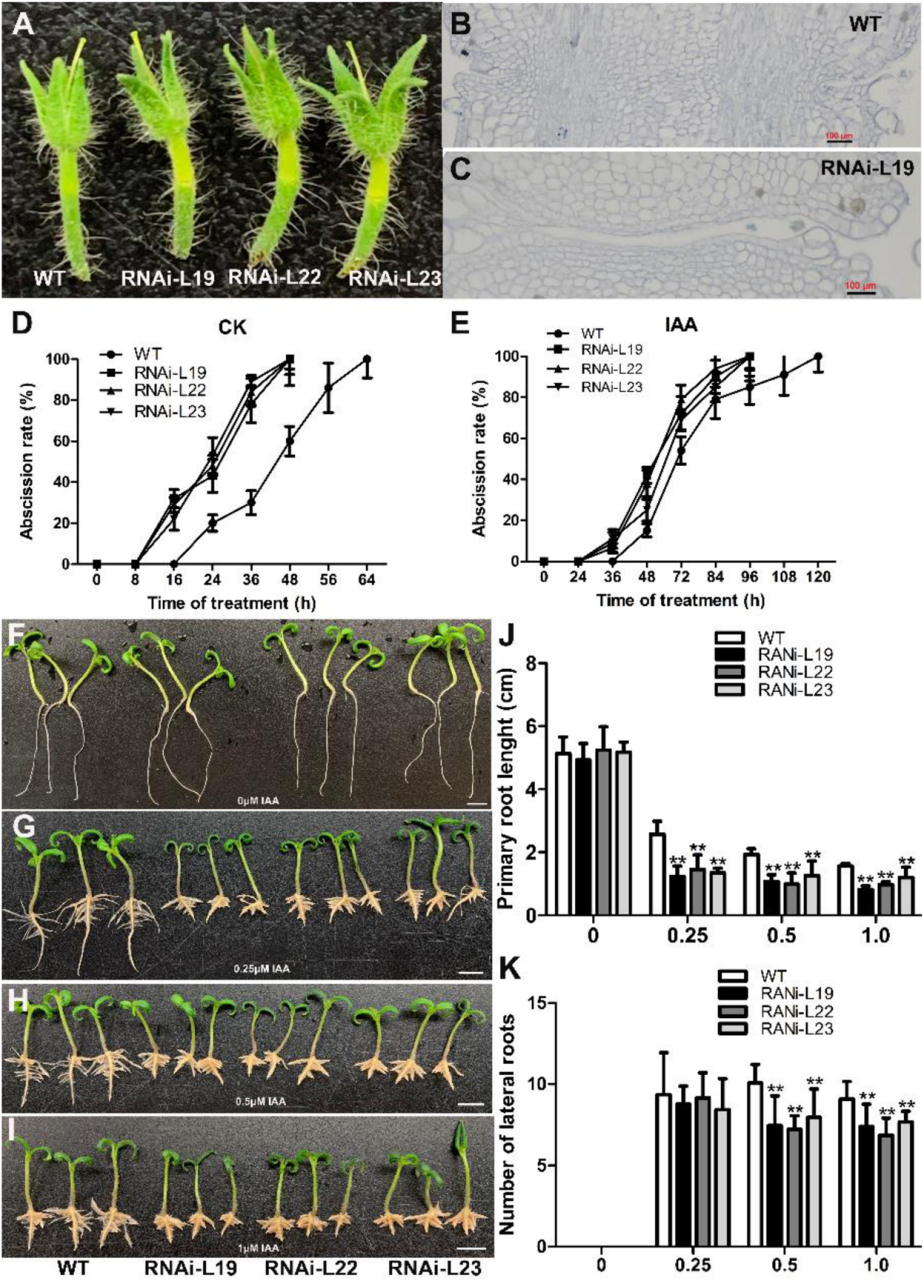
Effects of exogenous auxin on *SlBL4* RNAi plants. **A** Phenotypes of flower droppings after emasculation at 10 days. **B**, **C** Microsection of a 10-day floral pedicel abscission zone after emasculation in RNAi plants and WT tomato. **D**, **E** Abscission rate; **D** Timing of floral abscission-zone explants of tomato flowers following exposure to 1/2 MS; **E** Timing of floral abscission-zone explants of tomato flowers following exposure to 1/2 MS with 50 μg/g IAA. **F**–**I** Root development in WT plants and three independent *SlBL4* RNAi lines (L19, L22, and L23) assessed in two-week-old seedlings grown on 1/2 MS medium containing different concentrations of IAA (0, 0.25, 0.5, and 1.0 μM). **J** The lengths of primary roots in the *SlBL4* RNAi lines (L19, L22, and L23). **K** The lateral root numbers in the *SlBL4* RNAi lines (L19, L22, and L23); WT wild type, RNAi-L19, RNAi-L22, and RNAi-L23 three different lines of *SlBL4* RNAi plants. The asterisks indicate significant differences at *P* < 0.01 (**) as determined by the *t*-test

In emasculated flowers, the pedicel abscised at 10 and 14 days after emasculation in the RNAi lines and WT plants, respectively (Fig. 2A). Anatomic analysis of the floral pedicel AZ at 10 days after emasculation showed that the unpollinated flowers were abscised in the AZ in the *SlBL4* RNAi lines, but the cells began to separate at the AZ in the WT plants (Fig. 2B, C).

Sexton and Roberts reported that auxin and ethylene participate in the regulation of abscission in dicotyledonous plants^36^. To analyze the effect of IAA on RNAi line flower abscission, flowers were removed and replaced by 1/2 MS containing 50 μg/g IAA. The explants began to abscise at 8 h in the RNAi lines but at 16 h in the WT plants without IAA treatment, whereas they began to abscise at 24 h in the RNAi lines but at 36 h in the WT plants after IAA treatment (Fig. 2D, E). These results demonstrated that the RNAi lines exhibited an early-abscission phenotype and that IAA treatment could rescue this phenotype. For ethylene (20 μL/L) treatment of the explants, there was no difference between the RNAi lines and the WT plant in terms of the abscission rate (Fig. S1).

To investigate whether the downregulation of *SlBL4* alters auxin sensitivity, the root phenotype was further investigated. The RNAi lines exhibited shorter primary roots after IAA treatment compared with those of the WT seedlings. Lower lateral root numbers were observed in the RNAi lines after 0.5 and 1.0 μM IAA treatment compared with those of the WT seedlings (Fig. 2F–K). There was no difference between the RNAi lines and WT plant seedlings treated with 1 μM ACC for 7 days in the dark culture chamber (data not shown). These results suggested that the *SlBL4* RNAi tomato plants were sensitive to auxin in terms of root growth.

**Fig. 3:**
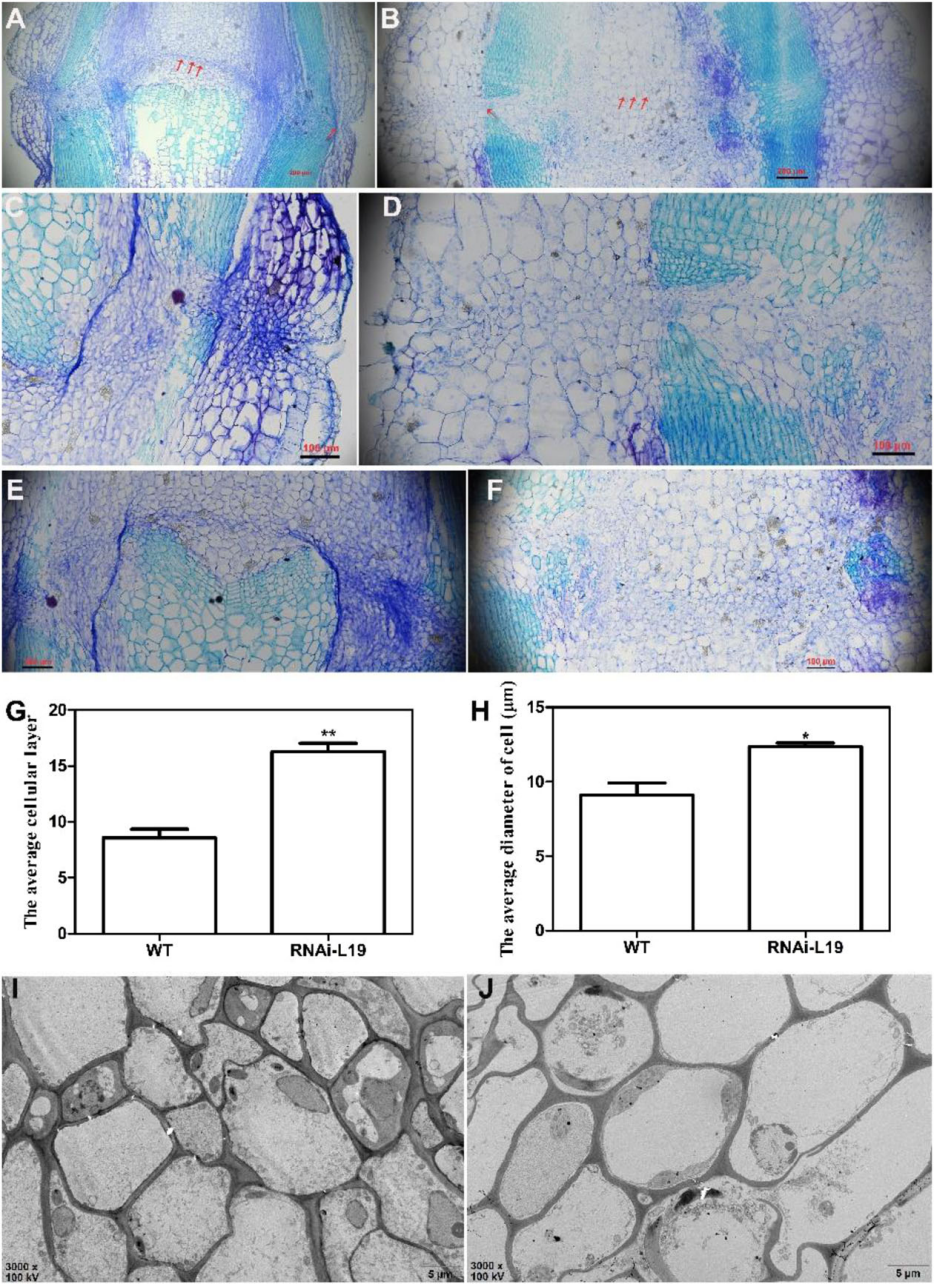
Anatomic analysis of the fruit pedicel abscission zone of the *SlBL4* RNAi plant. **A**–**F** Cross-sections of the fruit pedicel abscission zone at the 25 dpa stage as revealed by toluidine blue staining. **A**, **C**, **E** WT; **B**, **D**, and **F** *SlBL4* RNAi-L19. **G**, **H** The epidermal cell layers per cell in the fruit pedicel abscission zone of 25 dpa fruit pedicels in WT and *SlBL4* RNAi-L19 plates; **H** The epidermal diameter per cell in the fruit pedicel abscission zone of 25 dpa fruit pedicels in WT and *SlBL4* RNAi-L19 plants. **I**, **J** Transmission electron micrographs of the fruit pedicels of *SlBL4* RNAi-L19 and WT tomato at 25 dpa; **I** WT; **J** *SlBL4* RNAi-L19; scale bars: 5 μm; dpa day post anthesis, WT wild type

**Fig. 4:**
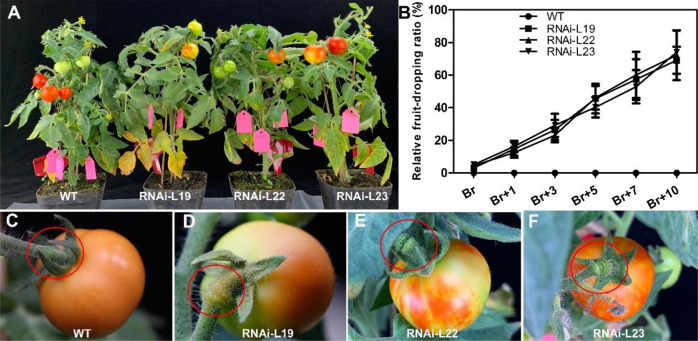
The fruit-dropping phenotypes of the *SlBL4* RNAi plants. **A**, **C**–**F** The fruit-dropping phenotypes. **B** Fruit-dropping statistical data. WT wild type,RNAi-L19, RNAi-L22, and RNAi-L23 three different lines of *SlBL4* RNAi plants

Anatomic analysis of the floral pedicel AZ showed that no obvious AZ formed, and more epidermis cell layers were observed in the *SlBL4* RNAi lines than in the WT (Fig. 3A–F). The epidermal cell layers and cell diameter were obviously increased by 90% and 36%, respectively, in the floral pedicel AZ of the *SlBL4* RNAi lines compared with the WT plants (Fig. 3G, H). Scanning electron microscopy (SEM) observations showed that epidermal cells were enlarged in the *SlBL4* RNAi lines compared with the WT (Fig. 3I, J). Silencing *SlBL4* positively affected the formation of pedicels in tomato plants. At the break (Br) stage, the fruit began to drop earlier in the *SlBL4* RNAi lines than in the WT plants (Fig. 4A and C–F). The ratio of fruit abscission was significantly increased on different Br days in *SlBL4* RNAi plants, whereas no fruit dropping was observed in the WT plants at the same stage (Fig. 4B). These observations indicated that the suppression of *SlBL4* activated pedicel abscission.

### RNA-seq and DEG analyses of pedicels of *SlBL4* RNAi lines and wild-type plants

**Fig. 5:**
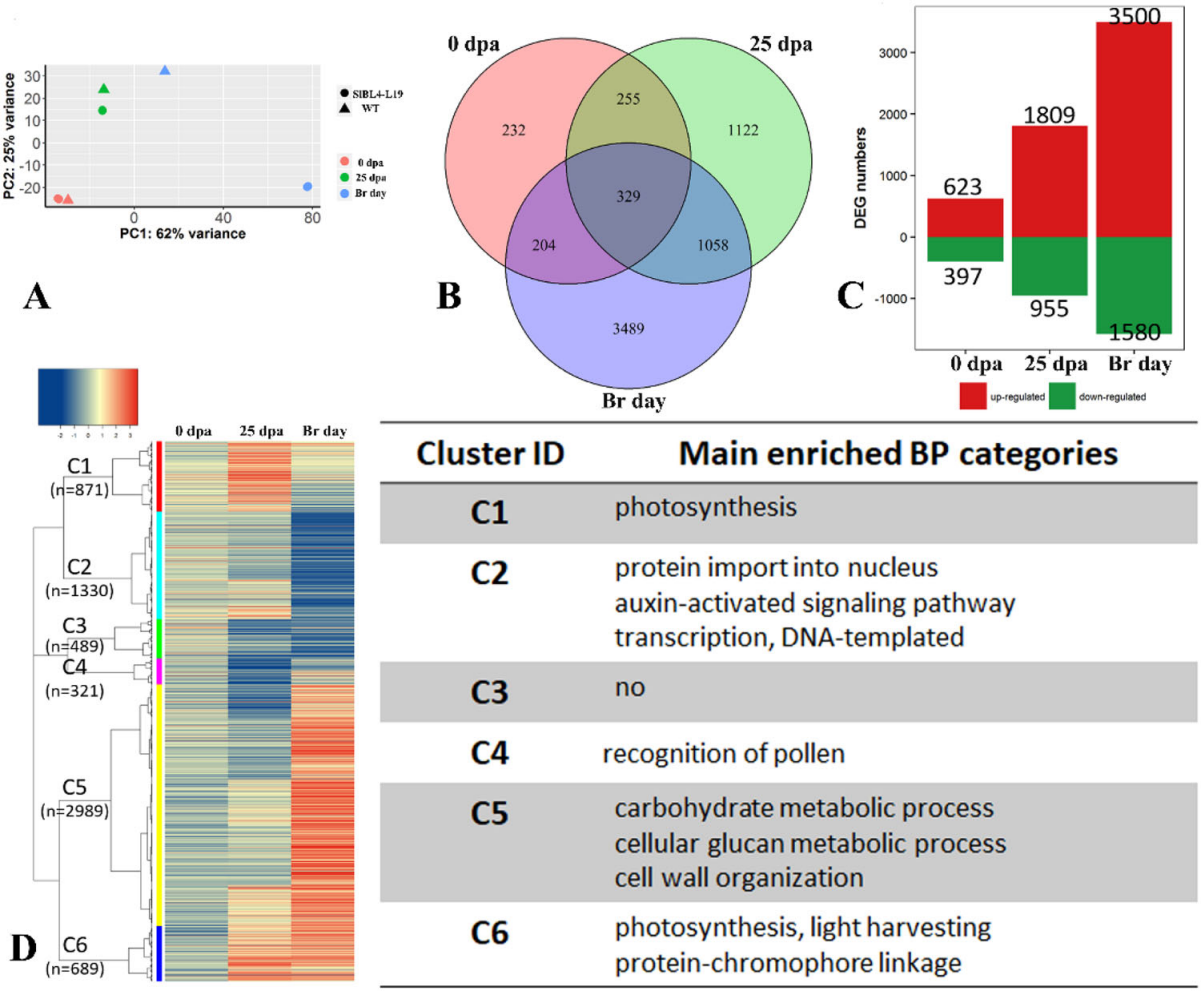
The DEG analysis of SlBL4 RNAi line 19 compared with wild-type tomato. **A** Principal component analysis (PCA) of gene expression data from the RNA-seq libraries. **B** Venn diagram of the differentially expressed genes (DEGs) between *SlBL4* RNAi and wild-type plants at three different fruit pedicel stages. **C** Upregulated (red bar) and downregulated (green bar) DEGs between *SlBL4* RNAi and wild-type plants at the three developmental stages of fruit pedicel. **D** Cluster analysis of DEGs at the three developmental stages of fruit pedicel

RNA-seq was performed to investigate the transcriptional mechanisms underlying the phenotype of thickened and pre-abscission pedicels in the *SlBL4* RNAi-L19 plant. DEGs (differentially expressed genes) were obtained for *SlBL4* RNAi-L19 plants compared with the WT plant in the AZ at the AS, 25 dpa, and Br stages. Correlation analysis and PCA demonstrated that the RNA-seq data for the RNAi-L19 plant pedicel samples were clearly differentiated from those for the WT plants at the 25 dpa and Br stages with good repeatability (Fig. S2 and Fig. 5A). The DEGs were categorized into three major classes, namely, cellular components, molecular functions, and biological processes, by GO annotation (Fig. S3A–C). KEGG pathway analysis showed that the DEGs were involved in metabolism, genetic information processing, and organismal systems (Fig. S3D–F). Under the criterion of a FDR ≤ 0.001, 329 DEGs were commonly upregulated or downregulated at the three developmental stages of tomato (Fig. 5B). In *SlBL4* RNAi AZs, 623, 1809, and 3500 genes were upregulated, and 397, 955, and 1580 genes were downregulated, respectively, at the anthesis day, 25 dpa, and Br stages compared with the WT plant (Fig. 5C). Cluster analysis showed that the DEGs were associated with the auxin-activated signaling pathway, the cellular glucan metabolic process, cell wall organization, light harvesting, photosynthesis, and protein-chromophore linkage (Fig. 5D and Tables S2–7).

### SlBL4 regulates the small cells of pedicel AZs

The silencing of *SlBL4* caused enlargement of the epidermal cells and disappearance of small cells at the separation zone compared with the WT plant (Fig. 3). In the transcriptomic analysis, the expression of *wuschel* (*WUS*), *cup-shaped cotyledon 2* (*CUP2*), *JOINTLESS* and *ovate family* proteins was decreased significantly, and *Bl*, *MYB transcription factor* (*MYB78*), and *LOB domain protein 1* (*LBD1*) were substantially upregulated in the *SlBL4* RNAi line compared with the WT plant at the abscission stage (Table 1). *WUS* (Solyc02g083950), *CUP* (Solyc07g06284), *JOINTLESS* (Solyc11g010570), and *OVATE* (Solyc02g085500) were downregulated by 4.3-, 1.77-, 1.09-, and 2.17-fold, and *Bl* (Solyc11g069030), *MYB78* (Solyc05g053330), and *LBD1* (Solyc11g072470) were upregulated by 4.57-, 3.39-, and 4.8-fold, respectively. Thus, these seven TF genes were likely involved in the regulation of abscission onset in the *SlBL4* RNAi plant. These genes were vital for maintaining undifferentiated cells in the AZ of tomato.

**Table 1:** Differentially expressed genes in the fruits of wild type and *SlBL4* RNAi plants at three stages of development

Gene ID	Annotation	Fold change (log2)			*p*-value		
		0 d	25 d	Br	0 d	25 d	Br
*Transcription factor*							
Solyc02g083950	Wuschel (WUS)	−1.41	−1.42	−4.30	<0.001	<0.001	<0.001
Solyc07g062840	Protein CUP-SHAPED COTYLEDON 2 (CUP)	−0.42	−0.40	−1.77	0.113	0.285	<0.001
Solyc11g010570	JOINTLESS	0.05	0.40	−1.09	0.747	<0.001	<0.001
Solyc02g085500	Ovate family protein	0.13	0.26	−2.17	0.201	0.002	<0.001
Solyc11g069030	MYB transcription factor (BLIND)	−1.03	−0.03	4.57	0.007	0.977	<0.001
Solyc05g053330	MYB transcription factor (MYB78)	−0.83	−1.4	3.39	<0.001	<0.001	<0.001
Solyc11g0 72470	LOB domain protein 1	−1.35	−0.54	4.80	<0.001	0.063	<0.001
Solyc05g010000	IDA	−1.34	0.000	7.656	0.262	1.000	<0.001
*Auxin-related*							
Solyc12g005310	Auxin responsive GH3 gene family (GH3-15)	0.91	3.11	5.88	0.072	<0.001	<0.001
Solyc01g107390	Auxin responsive GH3 gene family (GH3-2)	0.11	−1.97	2.63	0.819	<0.001	<0.001
Solyc02g064830	Indole-3-acetic acid-amido synthetase 3-3	−1.46	−1.24	2.48	<0.001	<0.001	<0.001
Solyc08g068490	Probable indole-3-acetic acid-amido synthetase GH3.5	1.26	1.10	−0.93	<0.001	<0.001	0.008
Solyc05g006220	IAA-amino acid hydrolase	0.06	−1.26	0.55	0.801	<0.001	<0.001
Solyc06g073060	IAA-amino acid hydrolase 6	0.17	0.81	1.56	0.164	<0.001	<0.001
Solyc10g079640	IAA-amino acid hydrolase 9	0.03	1.31	2.04	0.909	<0.001	<0.001
Solyc01g096340	SAUR family protein (SAUR2)	−0.24	1.38	0.61	0.029034	<0.001	<0.001
Solyc01g110560	SAUR3	0.61	0.95	1.12	0.064	0.001	<0.001
Solyc02g062230	SAUR32	−0.33	−0.81	1.78	0.520	0.117	<0.001
Solyc07g042490	SAUR33	0.35	0.57	1.73	0.018	<0.001	<0.001
Solyc03g082510	SAUR35	0.64	0.58	2.10	0.009	0.006	<0.001
Solyc03g082520	SAUR36	−0.28	0.00	3.20	0.339	1.00	<0.001
Solyc03g082530	SAUR37	−0.20	−1.85	4.34	0.535	<0.001	<0.001
Solyc03g097510	SAUR38	0.00	−0.68	6.59	1.000	0.483	<0.001
Solyc03g033590	SAUR50	0.25	−0.88	1.58	0.262	0.0076	<0.001
Solyc04g081270	SAUR52	0.76	3.41	5.11	0.211	<0.001	<0.001
Solyc05g05643 0	SAUR56	−0.17	−0.11	2.22	0.739	0.887	<0.001
Solyc05g056440	SAUR57	0.16	−0.19	2.53	0.829	0.798	<0.001
Solyc06g07265 0	SAUR61	0.00	−1.10	2.05	1.000	<0.001	<0.001
Solyc07g014620	SAUR63	0.00	0.16	1.56	1	0.671	<0.001
Solyc09g009980	SAUR70	−1.24	1.09	3.52	0.0294	0.105	<0.001
Solyc10g018340	SAUR71	−1.09	−3.20	2.02	0.302	<0.001	<0.001
Solyc03g124020	SAUR72-like	1.64	0.85	2.59	0.145	0.451	0.004
Solyc10g052560	SAUR75	0.05	0.89	2.40	0.954	0.369	0.005
Solyc09g065850.2	Auxin-responsive protein IAA (IAA3)	−0.16	−1.30	−3.11	0.003	<0.001	<0.001
Solyc06g053840	IAA4	0.32	0.16	−2.31	0.002	0.100	<0.001
Solyc06g053830	IAA7	0.07	−0.46	−2.71	0.533	<0.001	<0.001
Solyc06g008590	IAA10	1.21	0.51	1.09	<0.001	0.061	0.035
Solyc12g096980	IAA11	1.50	−0.39	−1.40	0.003	0.260	0.029
Solyc09g064530	IAA12	0.78	−0.02	−1.61	<0.001	0.937	<0.001
Solyc04g076850.2	IAA9	0.41	0.29	−1.25	<0.001	<0.001	<0.001
Solyc01g097290	IAA16	0.17	0.45	−3.62	<0.001	<0.001	<0.001
Solyc08g021820	IAA21	−0.24	−0.48	−2.30	0.425	<0.001	<0.001
Solyc06g008580	IAA22	0.36	−1.61	−4.19	0.119	<0.001	<0.001
Solyc09g083280.2	IAA23	0.29	−1.52	−2.09	0.007	<0.001	< 0.001
Solyc09g083290	IAA24	−0.11	−0.94	−4.60	0.619	<0.001	<0.001
Solyc09g090910	IAA 25	0.85	−0.58	−2.81	<0.001	<0.001	<0.001
Solyc07g019450	IAA33	−0.60	2.82	5.20	0.674	0.003	<0.001
Solyc06g066020	IAA 36	1.78	0.09	−3.95	<0.001	0.717	<0.001
Solyc05g047460	Auxin Response Factor 7B	−0.66	−0.55	−1.23	<0.001	<0.001	<0.001
Solyc08g082630	Auxin Response Factor 9A	1.23	1.21	−1.32	0.002	<0.001	0.041151
Solyc07g042260	Auxin response factor (ARF19)	−1.02	0.12	−0.20	<0.001	0.120	0.017
Solyc11g013310	Auxin transporter-like protein 3 (LAX3)	−0.25	−1.53	−0.34	<0.001	<0.001	<0.001
Solyc01g111310	Auxin transporter-like protein 3 (LAX2)	0.70	0.30	−1.51	<0.001	<0.001	<0.001
Solyc03g118740	SlPIN1	0.64	0.37	−3.08	<0.001	<0.001	<0.001
Solyc02g037550	SlPIN-LIKES 3	0.25	−0.38	−2.79	<0.001	<0.001	<0.001
Solyc01g068410	SlPIN5	1.05	−0.20	−2.60	0.010	0.702	<0.001
Solyc06g059730	SlPIN6	0.54	0.65	−2.04	0.146	0.123	<0.001
Solyc10g080880	SlPIN7	0.31	0.51	−2.08	0.044	<0.001	<0.001
*Cell wall hydrolysis/modification*							
Solyc09g091430	Probable pectate lyase 8	0.08	1.54	6.90	0.278	<0.001	<0.001
Solyc02g075620	Probable pectinesterase 53	0.844	1.765	5.319	<0.001	<0.001	<0.001
Solyc07g064190	Pectinesterase 3	−1.078	0.625	4.501	<0.001	0.689	<0.001
Solyc07g064180	Pectin esterase (PME2.1)	1.455	2.704	4.271	<0.001	<0.001	<0.001
Solyc01g102350	Pectin acetylesterase 12	0.341	1.052	3.584	<0.001	<0.001	<0.001
Solyc11g005770	Pectinesterase	−0.39	0.18	1.98	<0.001	0.035	<0.001
Solyc02g067630	Polygalacturonase 1	−1.59	1.60	12.41	0.153	0.130	<0.001
Solyc02g067640	Polygalacturonase 2	−1.22	0.04	11.55	0.338	1.000	<0.001
Solyc12g019230	Polygalacturonase-like	−2.55	−0.44	7.50	0.001	0.778	0.000
Solyc04g015530	Dehiscence polygalacturonase	−1.91	−0.17	7.33	<0.001	0.895	<0.001
Solyc08g081480	Polygalacturonase-like protein	0.381	1.193	3.706	<0.001	<0.001	<0.001
Solyc02g080210	Polygalacturonase-2a	−0.32	2.29	2.24	0.800	<0.001	<0.001
Solyc12g096750	Polygalacturonase 4	−1.68	1.51	5.85	<0.001	<0.001	<0.001
Solyc12g096740	Polygalacturonase 5	−4.05	−1.6	8.57	<0.001	<0.001	<0.001
Solyc12g019180	Polygalacturonase 7	−0.49	1.24	6.8	0.758	0.170	<0.001
Solyc01g094970	Polygalacturonase family protein	−2.75	−2.40	4.41	<0.001	<0.001	<0.001
Solyc03g006700	Peroxidase	−2.19	1.06	3.87	<0.001	<0.001	<0.001
Solyc08g081620	Endo-1,4-beta-glucanase precursor (Cel1)	0.23	1.50	5.38	<0.001	<0.001	<0.001
Solyc09g010210	Endo-1,4-beta-glucanase precursor (Cel2)	0.38	0.50	4.91	<0.001	<0.001	<0.001
Solyc09g075360	Endo-1,4-beta-glucanase precursor (Cel4)	−0.29	0.84	2.67	<0.001	<0.001	<0.001
Solyc11g040340	Endo-1,4-beta-glucanase precursor (Cel7)	0.85	−0.25	2.07	<0.001	<0.001	<0.001
Solyc08g082250	Endo-1,4-beta-glucanase precursor (Cel8)	−0.05	0.18	2.00	0.238	<0.001	<0.001
Solyc07g009380	Xyloglucan endotransglycosylase (XTH2)	1.17	5.17	6.05	<0.001	<0.001	<0.001
Solyc03g093110	Xyloglucan endotransglycosylase (XTH3)	0.64	0.30	1.42	<0.001	0.170	<0.001
Solyc03g093120	Xyloglucan endotransglycosylase (XTH3)	0.20	0.01	3.06	0.24	0.990	<0.001
Solyc03g093130	Xyloglucan endotransglycosylase (XTH3)	0.00	0.52	1.73	1.000	0.062	<0.001
Solyc11g065600	Xyloglucan endotransglycosylase (XTH4)	1.21	1.10	1.52	<0.001	<0.001	<0.001
Solyc01g091920	Xyloglucan endotransglycosylase (XTH7)	0.87	2.85	1.98	<0.001	<0.001	<0.001
Solyc12g011030	Xyloglucan endotransglycosylase (XTH9)	0.73	0.19	4.89	<0.001	0.420	<0.001
Solyc07g056000	Xyloglucan endotransglycosylase (XTH10)	−0.49	−0.56	2.90	<0.001	<0.001	<0.001
Solyc12g017240	Xyloglucan endotransglycosylase (XTH11)	−0.10	0.33	1.00	0.29	0.001	<0.001
Solyc07g052980	Xyloglucan endotransglycosylase (XTH16)	0.51	1.27	0.46	<0.001	<0.001	<0.001
Solyc01g112000	Expansin-like protein precursor 1 (EXLA1)	−0.18	−0.62	2.69	0.034	<0.001	<0.001
Solyc10g086520	Expansin (EXPA6)	1.09	0.76	4.06	0.006	0.130	<0.001
Solyc06g005560	Expansin 9 (EXPA9)	0.64	0.52	2.84	<0.001	<0.001	<0.001
Solyc06g076220	Expansin18 (EXPA18)	0.63	0.41	2.71	<0.001	<0.001	<0.001
Solyc08g077910	Expansin 45 (EXPA45)	−0.852	0.041	9.25	0.540	0.995	<0.001
Solyc06g051800	Fruit ripening regulated expansin1 (EXP1)	0.69	−0.01	4.01	<0.001	0.955	<0.001
Solyc06g049050	Expansin (EXP2)	0.85	2.06	2.06	<0.001	<0.001	<0.001
Solyc04g081870	Expansin precursor (EXP11)	0.42	0.87	4.64	<0.001	<0.001	<0.001
Solyc01g090810	Beta-expansin precursor(EXPB1)	−0.26	−2.84	9.59	0.847	<0.001	<0.001
Solyc05g007830	Alpha-expansin 1 precursor	1.15	0.17	0.41	<0.001	0.004	<0.001

**Fig. 6:**
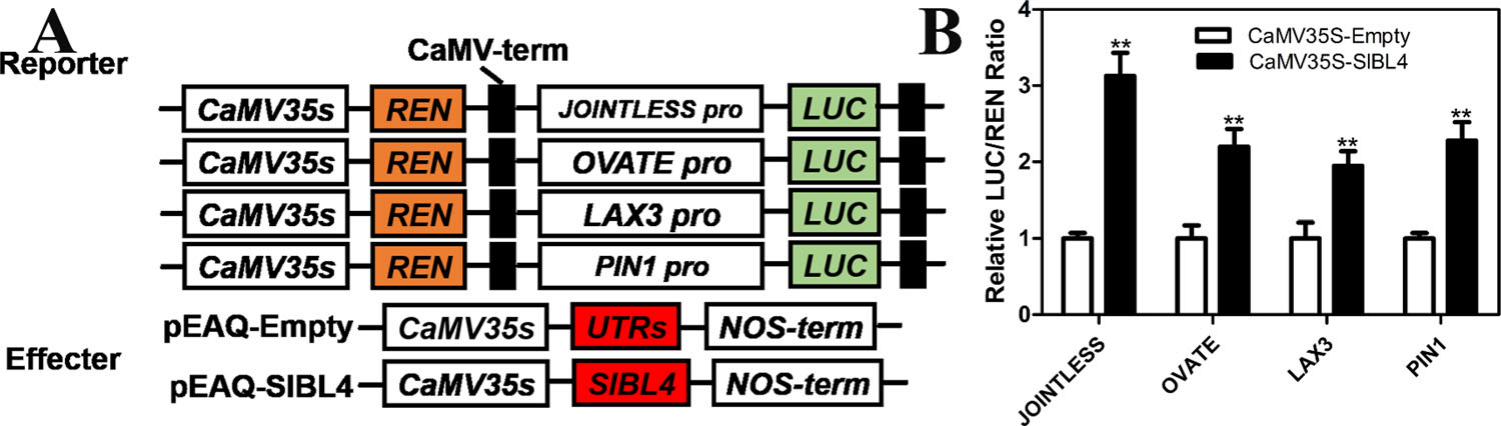
SlBL4 directly activates the expression of genes related to fruit pedicel development. **A** Diagram of the reporter and effector constructs used in the transient dual-luciferase assays in leaves of tobacco seedlings; LUC, firefly luciferase; REN, Renilla luciferase. **B** In vivo interactions of SlBL4 with the promoters obtained from the *JOINTLESS*, *OVATE, LAX3* or *PIN1* transient assays in tobacco leaves. The data are presented as the means (±SE), *n* = 6. Significant differences compared with the WT were determined by Student’s *t*-test: **P < 0.01

To examine the potential target genes of SlBL4 in fruit pedicel development, the promoter sequences were analyzed in *JOINTLESS* and *OVATE*, which revealed the SlBL4 binding (G/A) GCCCA (A/T/C) motif^29^. Transient dual-luciferase assays were performed to examine whether SlBL4 could directly activate or suppress the expression of the *JOINTLESS* and *OVATE* genes. Tobacco leaves were cotransformed with LUC reporter vectors driven by the promoters of the *JOINTLESS* and *OVATE* genes together with effector vectors carrying the CaMV35S promoter-driven *SlBL4* gene. Transient dual-luciferase assays showed that overexpression of SlBL4 significantly increased the luciferase activity driven by the promoters of *JOINTLESS* and *OVATE* compared with that of the empty control (Fig. 6A, B), indicating that SlBL4 activated the transcription of *JOINTLESS* and *OVATE*. The expression levels of *WUS*, *CUP*, and *Bl* were determined by RT-qPCR, and the results coincided with the RNA-seq results (Fig. S4).

### Silencing *SlBL4* affected the auxin level in AZ

Auxin plays critical role in the maintenance of fruit attachment to plants^3^. There were 103 auxin-related DEGs in the *SlBL4* RNAi line compared with the WT plant at the pre-abscission AZs. This included auxin-responsive genes, such as *Aux*/*IAA*, *Gretchen Hagen 3* (*GH3*), *small auxin upregulated RNA* (*SAUR*), and *auxin response factor* (*ARFs*), and auxin transport-related genes, such as *PIN* (pin-formed protein) and *like auxin* (*LAX*) (Table 1).

For instance, *ARF7B* (Solyc05g047460), *ARF9A* (Solyc08g082630), and *ARF19* (Solyc07g042260) were downregulated by 1.23-, 1.32-, and 0.2-fold, respectively, in the *SlBL4* RNAi line compared with the WT plant at the abscission stage. *LAX2* (Solyc01g111310) and *LAX3* (Solyc11g013310) were downregulated by 0.34- and 1.51-fold, respectively, in the *SlBL4* RNAi line compared with the WT plant at the abscission stage. *SlPIN1* (Solyc03g118740), *SlPIN-likes 3* (Solyc02g037550), *SlPIN5* (Solyc01g068410), *SlPIN6* (Solyc06g059730), and *SlPIN7* (Solyc10g080880) were downregulated by 3.08-, 2.79-, 2.60-, 2.04-, and 2.08-fold, respectively, in the *SlBL4* RNAi line compared with the WT plant at the abscission stage (Table 1). The promoter sequences were also analyzed in *LAX3* and *SlPIN1*, which revealed the SlBL4 binding (G/A) GCCCA (A/T/C) motif. Transient dual-luciferase assays showed that overexpression of SlBL4 significantly increased the luciferase activity driven by the promoters of *LAX3* and *SlPIN1* compared with that of the empty control (Fig. 6A, B), indicating that SlBL4 activated the transcription of *LAX3* and *SlPIN1*.

**Fig. 7:**
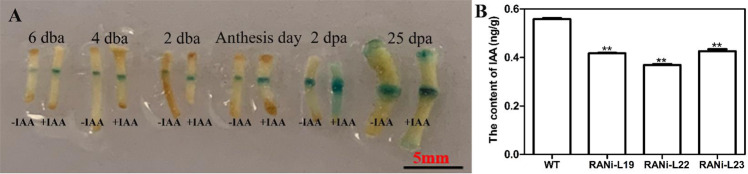
Effects of IAA treatment on the floral pedicel abscission zone of *SlBL4*pro::*GUS* plants and auxin concentrations in pedicel AZs. **A** Effects of exogenous auxin (+IAA) and control treatment (-IAA) on the floral pedicel abscission zones of *SlBL4*pro::*GUS* plants; 6 dba, 4 dba, 2 dba, anthesis day, 2 dpa, 25 dpa; dba: day before anthesis; dpa: day post anthesis. **B** The content of IAA; standard errors are indicated by vertical bars. The asterisks indicate significant differences at *P* < 0.01 (**) as determined by the *t*-test; WT wild type, RNAi-L19, RNAi-L22, RNAi-L23 three different lines of *SlBL4* RNAi plants

To analyze the effect of IAA on the floral pedicel AZ of *SlBL4*pro::*GUS* plants, the flower was removed and replaced by 1/2 MS medium containing 50 μg/g IAA for 24 h, which accelerated *GUS* expression in the pedicel AZ compared with that in the control (-IAA) (Fig. 7A). In addition, we examined the IAA concentrations in tomato pedicel AZs by acquisition ultraperformance liquid chromatography. The IAA concentrations in the AZs of *SlBL4* RNAi plants were lower than those in the AZs of WT plants (Fig. 7). The expression of *ARF9A* and *SlPIN7* was determined by qRT-PCR, and the results coincided with the RNA-seq results (Fig. S4). In conclusion, silencing *SlBL4* influenced the auxin efflux, signaling, and content in tomato pedicels.

### Suppression of *SlBL4* induces the expression of genes encoding cell wall hydrolytic enzymes

The expression of genes encoding cell wall degrading and remodeling enzymes, including pectinases (PG, PL), cellulase (Cel), xyloglucan endotransglucosylase-hydrolase (XTH), and expansin (EXP), was reported to be induced in response to the abscission stimulus. Our transcriptome analyses showed that many of the genes mentioned above were expressed at higher levels in the *SlBL4* RNAi line than in the WT plant at the abscission stage. Among the DEGs, 16 *PG/PL* genes, 5 *Cel* genes, 10 *XTH* genes, 10 *EXP* genes, and 1 *peroxidase* (*POD*) gene were found (Table 1). For instance, *PG1* (Solyc02g067630), *PG2* (Solyc02g067640), and *PE* (Solyc11g005770) were upregulated by 12.41-, 11.55-, and 1.98-fold, respectively. *Cel1* (Solyc08g081620) was upregulated by 5.38-fold. *XTH2* (Solyc07g009380) was upregulated by 6.05-fold. *EXPA45* (Solyc08g077910) and *EXPB1* (Solyc01g090810) were upregulated by 9.25- and 9.59-fold, respectively. These genes were vital for cell wall remodeling and middle lamella degradation at the late stages of the abscission process. The expression levels of *PG1*, *PE*, *XTH2*, and *EXPA45* were evaluated by RT-qPCR, and the results coincided with the RNA-seq results (Fig. S4).

## Discussion

BELL TFs play various roles in plant morphology and fruit development^29,37,38^, whereas they are seldom reported to be involved in fruit pedicel organogenesis and abscission. Here, we reported that SlBL4 is an important regulator of fruit pedicel organogenesis and abscission in tomatoes.

### SlBL4 regulated *WUS*, *Bl*, *CUP*, *OVATE*, and *Ls* in the regulation of competency to respond to abscission-promoting signaling

Abscission can occur at four key steps, namely, differentiation of the AZ, acquisition of the competence to respond to abscission signals, activation of organ abscission, and formation of a protective layer on the main body side of the AZ^6,7,39^. We elucidated the role of SlBL4 in abscission by observing the *SlBL4* RNAi fruits at 25 dpa. Our anatomic analysis of the floral pedicel AZ showed greater enlargement of the epidermal cells and disappearance of the small cells at the separation zone in the *SlBL4* RNAi plant pedicel compared with the control (Fig. 3). These results suggested that the size and proliferation of the AZ cells are likely to be activated due to the repression of *SlBL4* expression. The involvement of TF genes, such as *WUS*, *Bl*, *CUP*, *JOINTLESS*, *OVATE*, *LBD1*, and *Ls*, was evident in the anthesis pedicel AZs^14,40–42^. Moreover, our analyses also revealed AZ-specific downregulation of *WUS*, *CUP*, *JOINTLESS*, and *OVATE* and upregulation of *Bl*, *MYB78*, and *LBD1* in the *SlBL4* RNAi line compared with the WT plant at the abscission stage (Table 1). WUS, OVATE, and CUP function coordinate to maintain cells in an undifferentiated state and to maintain a small cell size, which are critical for meristem activities^7,40,42,43^. Therefore, the reduced expression of *WUS*, *CUP*, *JOINTLESS*, and *OVATE* caused by an abscission signal may have resulted in enlargement of the separation zone cells for the onset of abscission in the RNAi line compared with the WT plant. Our results indicated that SlBL4 directly activated *JOINTLESS* and *OVATE* expression, thus accounting for the disappearance of the small cells at the separation zone in the *SlBL4* RNAi plant pedicel (Fig. 6). The *Bl* gene, which encodes the R2R3 class MYB TF, controls lateral meristem development^17,44^ and is upregulated in the AZ-formation stage in tomato^14^. In combination with the *SlBL4* expression pattern in AZs, this finding explains why SlBL4 contributes to the maintenance of the undifferentiated status of cell proliferation and differentiation of flower pedicel AZs by affecting the expression of meristem activity genes.

### SlBL4 regulates the auxin gradient in the pedicel and affects the expression of auxin transport-related genes

Several studies have found that auxin plays a critical role in pedicel abscission via continuous flow from flowers or fruit^3,8,10,43^. Our results demonstrated that silencing *SlBL4* affects the formation of pedicels in tomato plants. The IAA content in the RNAi lines was less than that in the WT plants (Fig. 7B). Furthermore, IAA treatments were performed on the seeding and floral pedicel AZs to show their effects on the abscission process. The results showed that the explants began to abscise at 8 h in the RNAi lines not treated with IAA (Fig. 2D), but they began to abscise at 24 h in the RNAi lines treated with IAA. Compared with this, the explants began to abscise at 16 h in the WT plants not treated with IAA (Fig. 2E). The root growth phenotype also indicated that the RNAi lines were more sensitive to IAA treatment than the WT plants (Fig. 2J and K). All of the above results demonstrated that IAA could postpone AZ abscission and that knockdown of *SlBL4* could cause an early abscission phenotype by reducing the accumulation of IAA. At the break stage, the fruit began to drop, and the auxin contents were reduced in the AZs of the *SlBL4* RNAi lines compared with the WT plants, suggesting that *SlBL4* plays a role in modulating auxin levels (Figs. 4 and 7). The effect of IAA-related gene expression can also help clarify which genes are likely to directly or indirectly participate in the abscission process. SlPIN1 plays an important role in regulating not only the basipetal auxin flux from the seed to the plant but also the fruit to the basal organ^3,45–47^. Silencing the expression of SlPIN1 decreases the auxin content in the AZ, which is necessary for preventing tomato pedicel abscission^46^. In *A. thaliana*, LAX3 has been shown to actively regulate auxin influx^48,49^. *LAX3*, which is an ortholog of *AtLAX3*, plays a role in the regulation of auxin influx in the tomato pericarp^47^. *SlPIN1 and LAX3* were downregulated by 3.08- and 0.34-fold, respectively, in the *SlBL4* RNAi line compared with the WT plant at the abscission stage (Table 1). Our results also indicated that SlBL4 directly activated the expression of the auxin efflux transporter genes *SlPIN1* and *LAX3*, thus accounting for the lower auxin content in the *SlBL4* RNAi plant pedicel (Figs. 6–7). *ARF7* and *ARF19* are involved in abscission in *A. thaliana*^50^, and the expression of *SlARF19* is upregulated in flower AZs (FAZs)^51^. The expression level of ARF9 homolog (ARF9A) was shown to be significantly higher in the proximal region than in AZs and distal regions^10^. Our transcriptomic results showed that the transcript abundances of three auxin response genes (ARFs; ARF7B, ARF9A, or ARF19) were downregulated in the RNAi line compared with the WT plant at the abscission stage (Table 1). Constant auxin flux plays an important role in preventing abscission^1^. *SlPIN9* and *PIN-like 3* were downregulated in the FAZs of *KD1* antisense tomato plants, which suggested that the *KD1* gene plays a role in manipulating auxin levels by altering the expression of auxin efflux transporters^33^. The *SlPIN3*, *SlPIN5*, *SlPIN6*, and *SlPIN7* genes were highly expressed in FAZs^52^. Five PIN genes (*SlPIN1*, *SlPIN-LIKES 3*, *SlPIN5*, *SlPIN6*, and *SlPIN7*) and two *LAX* genes (*LAX2* and *LAX3*) were downregulated in the *SlBL4* RNAi line compared with the WT plant at the abscission stage (Table 1), indicating a complex interplay among different components (AUX/IAA, ARFs, PIN, and LAXs) of the auxin response pathway during tomato pedicel abscission. These results were consistent with our hypothesis that *SlBL4* plays a role in manipulating auxin levels in the AZ, perhaps by regulating transport genes for auxin influx and efflux. The timing of pedicel abscission was determined by the auxin level^43^. Therefore, *SlBL4* may be involved in the timing of abscission onset by regulating the expression of ARFs, auxin influx, and efflux transport genes.

### The *SlBL4* gene suppresses cell wall hydrolytic enzymes in tomato pedicles

The last step of abscission is an activation of cell wall-degrading enzymes such as PG, Cel, XTH, and EXP and subsequent removal of plant organs^43–61^. In our study, 8 *polygalacturonase*, 5 *Cel*, *10 XTH*, *10 EXP*, and 1 *peroxidase* gene were upregulated in the *SlBL4* RNAi line compared with the WT plants at the abscission stage (Table 1). Silencing of PGs delayed abscission in tomato^62^. *PG1* was highly expressed in flower AZs, and its expression was inhibited by auxin^63,64^. Cel1 and Cel2 play important roles in tomato flower and leaf abscission^53,65^. In our previous study, we showed that *SlPE* expression was regulated by SlBL4, that the transcription of *SlPE* was directly repressed by SlBL4, and that such actions were involved in pectin depolymerization^29^. The expression of *SlPE* was also increased in the *SlBL4* RNAi line compared with the WT plant at the abscission stage, which suggested that *SlPE* is also involved in cell wall modification and cell separation during pedicel abscission in tomato. Expansins (EXPs) are involved in the cell wall and pectin modification during the abscission process^66–70^. Expansins also reportedly regulate pedicel abscission in *A. thaliana* and soybean (*Glycine max* L.) and leaflet abscission in elderberry (*Sambucus nigra*), tomato, and citrus (*Citrus* L.)^52,60,66,68,71^. We observed that the expression levels of several *EXP* genes, such as *EXPA45* or *EXPB1*, were significantly upregulated in the *SlBL4* RNAi line compared with the WT plant at the abscission stage.

In conclusion, SlBL4 plays a role in establishing and maintaining the properties of pre-abscission tomato pedicel AZs by regulating shoot meristem genes, auxin influx, and efflux transport genes and cell wall hydrolytic genes. The results of this study provide insight into a new aspect of the regulation of organ development and abscission by BELL family proteins with regard to tomato pedicel formation.

## Supplementary information

Supplementary information

Supplementary figure

## Data Availability

The data used to support the findings of this study are available from the corresponding author upon request.
