# Supplementary material for: Tomato SlBL4 plays an important role in fruit pedicel organogenesis and abscission
Source: Hortic Res. 2021 Apr 1;8:78. doi: 10.1038/s41438-021-00515-0 (PMC8012377; doi:10.1038/s41438-021-00515-0)
Supplement: Supplementary file 1 — Supplementary information [file 41438_2021_515_MOESM1_ESM.docx]

**Figure S1 Effects of exogenous ethylene on the abscission rate of *SlBL4* RNAi and WT plants.** (A) Timing of floral abscission-zone explants of tomato flowers following exposure to 1/2 MS. (B) Timing of floral abscission-zone explants of tomato flowers following exposure to 1/2 MS with 20 μL/L ethylene.

**
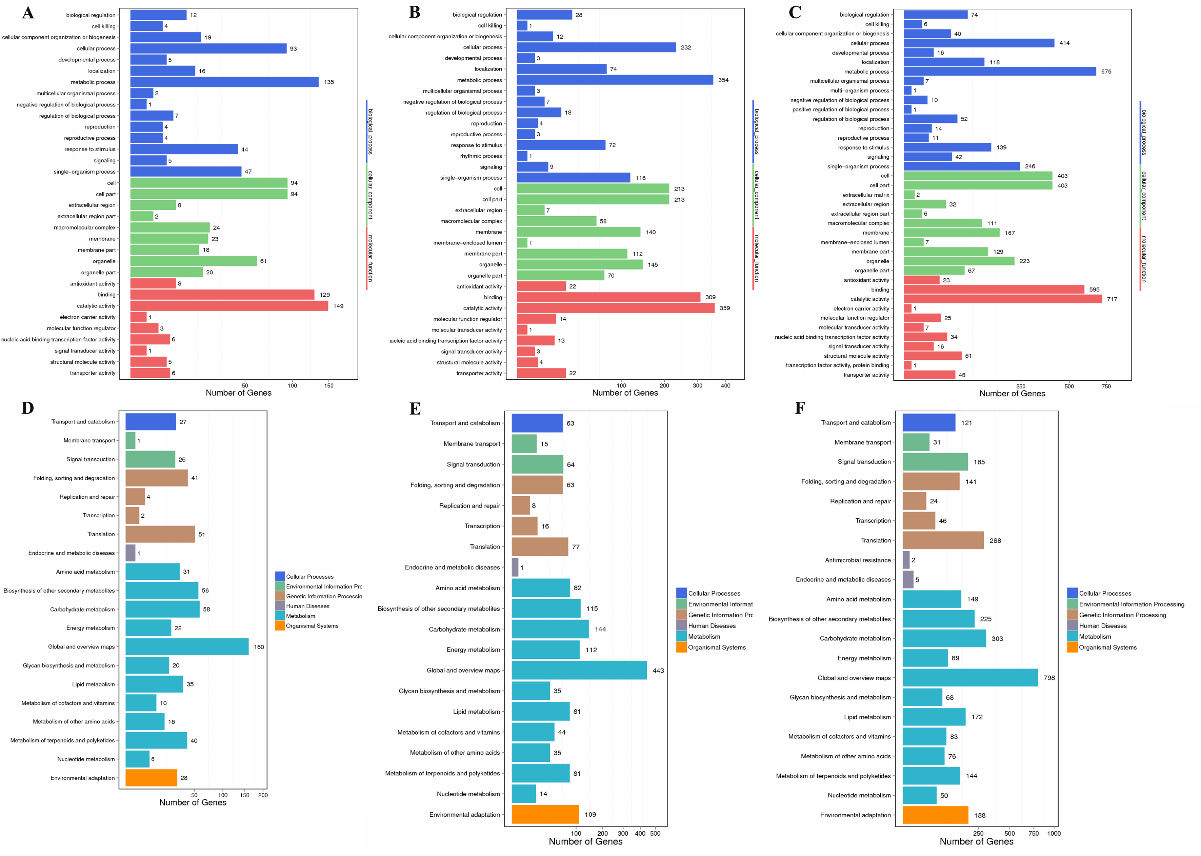
**
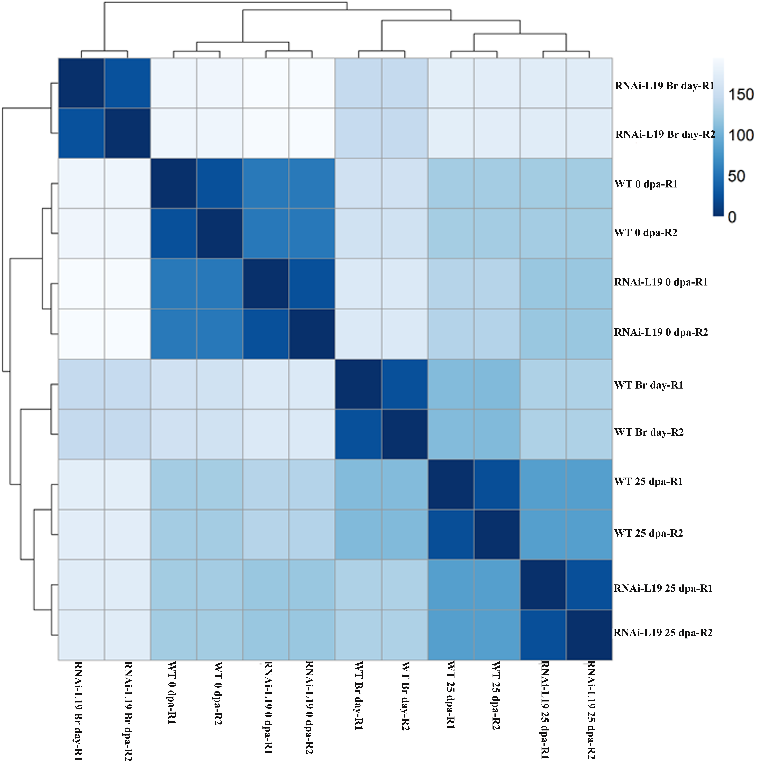
**Figure S2** **Transcriptomic data analysis of fruit pedicels in *SlBL4* RNAi and wild type plants at three developmental stages.** Pearson correlation matrix of gene expression data [log2(read counts + 1)] from all of the RNA-seq libraries. The distance matrix of gene expression from all of the RNA-seq libraries was implemented by DEseq2. The dendrograms were generated by hierarchically clustering samples based on correlation values transformed into distance values. The darker blue indicates a higher correlation.

**Figure S3** **Enrichment analyses of GO terms and KEGG pathways in the fruit pedicles of *SlBL4* RNAi plants compared with the wild type at three stages of development.**


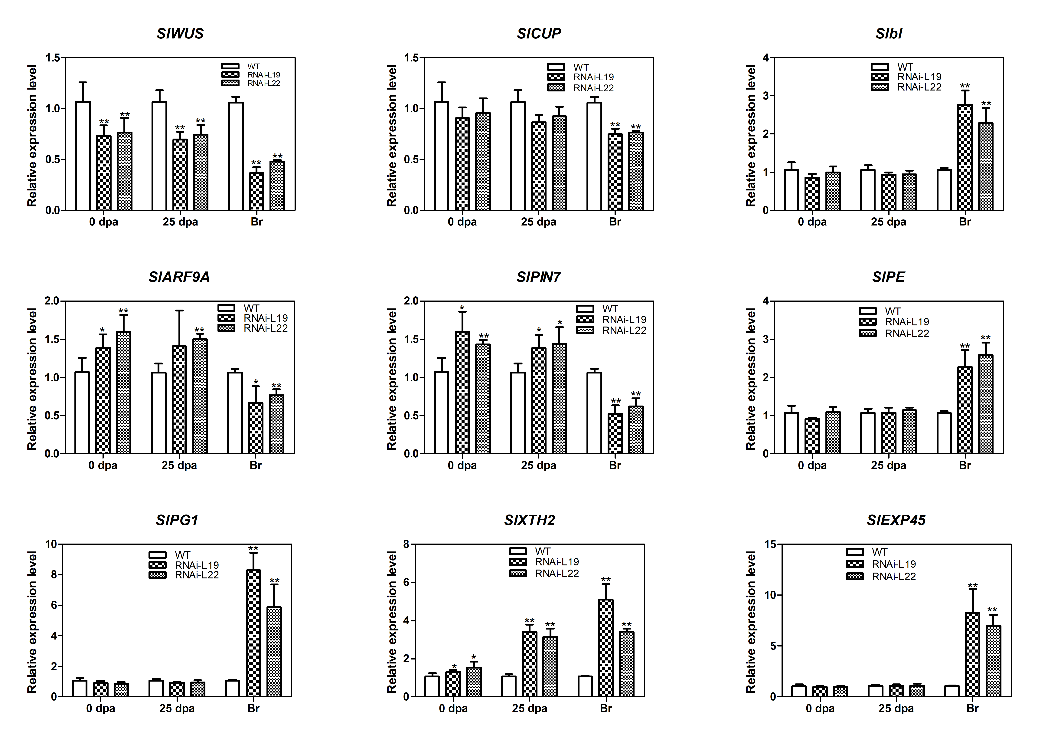
**Figure S4 qRT-PCR confirmation of RNA-seq DEGs between *SlBL4* RNAi line 19 and wild type tomato.** The data are presented as the mean values from three independent experiments. Standard errors are indicated by vertical bars. The asterisks indicate significant differences at 0.05 > P > 0.01 (*) or P < 0.01 (**) as determined by the *t*-test; WT: wild type; RNAi-L19, RNAi-L22, RNAi-L23: three different lines of *SlBL4* RNAi plants.

**Supplementary Table S1. Primer sequences**

| Primer | Primer sequence (5’ to 3’) | Orientation |
| --- | --- | --- |
| SlBL4-PF | GCGTCGACGGGATCTACTTATGTATTTGGC | Sense |
| SlBL4-PR | CGGGATCCGCCTATAACAAGAAAAATGTTCTA | Antisense |
| qSlBL4F | AGCAGTGGGCACTTTCAGCAGC | Sense |
| qSlBL4R | CTGGCAAGCCTCTTTGTGGCCT | Antisense |
| SlUbiF | GCCGACTACAACATCCAGAAGG | Sense |
| SlUbiR | TGCAACACAGCGAGCTTAACC | Antisense |
| SlWUS-F | GTGCAGGCAAAGTAGTAGCC | Sense |
| SlWUS-R | TGTTCAGCAGTTGGAGACCT | Antisense |
| qSlCUP-F | AATGGTGCCGCTACTTCAAC | Sense |
| qSlCUP-R | GCGGTAGTGCTATACGGAGA | Antisense |
| qSlBLF | TGGGAAGAGCTCCATGTTGT | Sense |
| qSlBL-R | CCACATCTCCTTAGTCCTGCT | Antisense |
| qSlPG1-F | TTTGGGCTTGCAAGAACTCC | Sense |
| qSlPG1-R | TTCCTGGAGCGGATACCTTT | Antisense |
| qSlPE-F | GGTGCTGATACCGGGTCATGG | Sense |
| qSlPE-R | TTTCAACCCGGTCGGCACTC | Antisense |
| qSlXTH2-F | AATGCTGAGGAATGGGCAAC | Sense |
| qSlXTH2-R | TTGGCTGTGGGATTCTTGGA | Antisense |
| qSlEXP45-F | AGTGACTGACAGTGGTGAGG | Sense |
| qSlEXP45-R | AACCACTCCCTTTGGGAACA | Antisense |
| qSlARF9A-F | ACCTTTGGCATCAACAGCAG | Sense |
| qSlARF9A-R | TACCGTCTTCTGGCTCATCC | Antisense |
| qSlPIN7-F | TGCTGCTAGTGTGATGACCA | Sense |
| qSlPIN7-R | CATTCCAAGACCAGCATCGG | Antisense |
| 0800-JOINTLESS -F | TATAGGGCGAATTGGGTACCCCACTCCATCTCACCATA | Sense |
| 0800-JOINTLESS -R | TTGGCGTCTTCCATGGGAGGGAACAAAGAATAAACC | Antisense |
| 0800- LAX3-F | TATAGGGCGAATTGGGTACCGGGTCTGAAGAGGTCTGGA | Sense |
| 0800-LAX3-R | TTGGCGTCTTCCATGGGGACGAAAGCATAGTGGTG | Antisense |
| 0800- PIN1-F | TATAGGGCGAATTGGGTACCTCAGAGAGAAGGGTGAGGG | Sense |
| 0800- PIN1-R | TTGGCGTCTTCCATGGTTGGTTGGAGGTGGTGTC | Antisense |
| 0800-ovate-F | TATAGGGCGAATTGGGTACCTGTGGGTGTTTATTACCA | Sense |
| 0800-ovate-R | TTGGCGTCTTCCATGGTAACTCTGGAGAACCGAA | Antisense |
| 62sk-SlBL4-F | TAGAACTAGTGGATCCATGGCAGAGCCTCTTCGG | Sense |
| 62sk-SlBL4-R | CGGTATCGATAAGCTTCTTCATCGTAAATAAATG | Antisense |

**Supplementary Table S2.** **List_C1**

| \| Category \| Overrepresented_p-value \| Underrepresented_p-value \| NumDEInCat \| NumInCat \| Term \| Ontology \| Padj \| \| --- \| --- \| --- \| --- \| --- \| --- \| --- \| --- \| \| GO:0009765 \| 1.24E-20 \| 1 \| 19 \| 33 \| Photosynthesis, light harvesting \| BP \| 1.24E-20 \| \| GO:0006364 \| 2.64E-14 \| 1 \| 28 \| 144 \| rRNA processing \| BP \| 2.64E-14 \| \| GO:0018298 \| 3.27E-14 \| 1 \| 17 \| 49 \| Protein-chromophore linkage \| BP \| 3.27E-14 \| \| GO:0010207 \| 8.14E-13 \| 1 \| 22 \| 98 \| Photosystem II assembly \| BP \| 8.14E-13 \| \| GO:0019344 \| 1.01E-12 \| 1 \| 18 \| 63 \| Cysteine biosynthetic process \| BP \| 1.01E-12 \| \| GO:0015979 \| 9.01E-12 \| 1 \| 21 \| 116 \| Photosynthesis \| BP \| 9.01E-12 \| \| GO:0009773 \| 4.39E-11 \| 1 \| 12 \| 29 \| Photosynthetic electron transport in photosystem I \| BP \| 4.39E-11 \| \| GO:0015995 \| 1.23E-10 \| 1 \| 16 \| 62 \| Chlorophyll biosynthetic process \| BP \| 1.23E-10 \| \| GO:0006098 \| 2.33E-10 \| 1 \| 21 \| 117 \| Pentose-phosphate shunt \| BP \| 2.33E-10 \| \| GO:0009637 \| 5.98E-09 \| 1 \| 12 \| 40 \| Response to blue light \| BP \| 5.98E-09 \| \| GO:0030003 \| 7.38E-09 \| 1 \| 10 \| 27 \| Cellular cation homeostasis \| BP \| 7.38E-09 \| \| GO:0009657 \| 7.56E-09 \| 1 \| 9 \| 21 \| Plastid organization \| BP \| 7.56E-09 \| \| GO:0070838 \| 1.25E-08 \| 1 \| 9 \| 22 \| Divalent metal ion transport \| BP \| 1.25E-08 \| \| GO:0010114 \| 3.92E-08 \| 1 \| 10 \| 30 \| Response to red light \| BP \| 3.92E-08 \| \| GO:0035304 \| 7.02E-08 \| 1 \| 13 \| 60 \| Regulation of protein dephosphorylation \| BP \| 7.02E-08 \| \| GO:0010218 \| 1.05E-07 \| 1 \| 10 \| 33 \| Response to far red light \| BP \| 1.05E-07 \| \| GO:0006636 \| 3.70E-07 \| 1 \| 9 \| 29 \| Unsaturated fatty acid biosynthetic process \| BP \| 3.70E-07 \| \| GO:0009697 \| 2.04E-06 \| 1 \| 9 \| 36 \| Salicylic acid biosynthetic process \| BP \| 2.04E-06 \| \| GO:0043900 \| 2.05E-06 \| 1 \| 6 \| 13 \| Regulation of multiorganism process \| BP \| 2.05E-06 \| \| GO:0019684 \| 2.12E-06 \| 1 \| 8 \| 29 \| Photosynthesis, light reaction \| BP \| 2.12E-06 \| \| GO:0019288 \| 2.39E-06 \| 0.999999 \| 17 \| 133 \| Isopentenyl diphosphate biosynthetic process, methylerythritol 4-phosphate pathway \| BP \| 2.39E-06 \| \| GO:0009595 \| 3.60E-06 \| 1 \| 6 \| 14 \| detection of biotic stimulus \| BP \| 3.60E-06 \| \| GO:0010103 \| 4.02E-05 \| 0.999994 \| 10 \| 61 \| Stomatal complex morphogenesis \| BP \| 4.02E-05 \| \| GO:0019761 \| 5.47E-05 \| 0.999993 \| 8 \| 40 \| Glucosinolate biosynthetic process \| BP \| 5.47E-05 \| \| GO:0010310 \| 9.66E-05 \| 0.999989 \| 7 \| 33 \| Regulation of hydrogen peroxide metabolic process \| BP \| 9.66E-05 \| \| GO:0019253 \| 0.000172 \| 0.999998 \| 3 \| 4 \| Reductive pentose-phosphate cycle \| BP \| 0.000172 \| \| GO:0010196 \| 0.000199 \| 0.999997 \| 3 \| 5 \| Nonphotochemical quenching \| BP \| 0.000199 \| \| GO:0019252 \| 0.000231 \| 0.999944 \| 12 \| 107 \| Starch biosynthetic process \| BP \| 0.000231 \| \| GO:0016117 \| 0.000345 \| 0.999934 \| 9 \| 64 \| Carotenoid biosynthetic process \| BP \| 0.000345 \| \| GO:0010027 \| 0.000369 \| 0.999893 \| 14 \| 144 \| Thylakoid membrane organization \| BP \| 0.000369 \| \| GO:0009862 \| 0.000384 \| 0.999945 \| 7 \| 41 \| Systemic acquired resistance, salicylic acid mediated signaling pathway \| BP \| 0.000384 \| \| GO:0000165 \| 0.000444 \| 0.999948 \| 6 \| 30 \| MAPK cascade \| BP \| 0.000444 \| \| GO:0050832 \| 0.000512 \| 0.999909 \| 8 \| 56 \| Defense response to fungus \| BP \| 0.000512 \| \| GO:0016114 \| 0.000556 \| 0.999968 \| 4 \| 13 \| Terpenoid biosynthetic process \| BP \| 0.000556 \| \| GO:0043085 \| 0.000731 \| 0.999862 \| 8 \| 58 \| Positive regulation of catalytic activity \| BP \| 0.000731 \| \| GO:0009902 \| 0.000741 \| 0.999841 \| 9 \| 73 \| Chloroplast relocation \| BP \| 0.000741 \| \| GO:0009814 \| 0.001025 \| 0.999967 \| 3 \| 7 \| Defense response, incompatible interaction \| BP \| 0.001025 \| \| GO:0009768 \| 0.00127 \| 1 \| 2 \| 2 \| Photosynthesis, light harvesting in photosystem I \| BP \| 0.00127 \| \| GO:0010200 \| 0.001407 \| 0.999788 \| 6 \| 38 \| Response to chitin \| BP \| 0.001407 \| \| GO:0000023 \| 0.001677 \| 0.999593 \| 9 \| 83 \| Maltose metabolic process \| BP \| 0.001677 \| \| GO:0032544 \| 0.001925 \| 0.999917 \| 3 \| 8 \| Plastid translation \| BP \| 0.001925 \| \| GO:0030154 \| 0.002224 \| 0.999559 \| 7 \| 54 \| Cell differentiation \| BP \| 0.002224 \| \| GO:0007000 \| 0.004164 \| 0.999744 \| 3 \| 10 \| Nucleolus organization \| BP \| 0.004164 \| \| GO:0010363 \| 0.005021 \| 0.998833 \| 7 \| 63 \| Regulation of plant-type hypersensitive response \| BP \| 0.005021 \| \| GO:0006612 \| 0.005185 \| 0.998788 \| 7 \| 63 \| Protein targeting to membrane \| BP \| 0.005185 \| \| GO:0031348 \| 0.005533 \| 0.998879 \| 6 \| 48 \| Negative regulation of defense response \| BP \| 0.005533 \| \| GO:0007623 \| 0.005848 \| 0.999287 \| 4 \| 22 \| Circadian rhythm \| BP \| 0.005848 \| \| GO:0009965 \| 0.006341 \| 0.998269 \| 8 \| 81 \| Leaf morphogenesis \| BP \| 0.006341 \| \| GO:0010155 \| 0.006673 \| 0.998853 \| 5 \| 35 \| Regulation of proton transport \| BP \| 0.006673 \| \| GO:0045038 \| 0.007459 \| 0.999817 \| 2 \| 4 \| Protein import into chloroplast thylakoid membrane \| BP \| 0.007459 \| \| GO:0006535 \| 0.009334 \| 0.999181 \| 3 \| 13 \| Cysteine biosynthetic process from serine \| BP \| 0.009334 \| \| GO:0009867 \| 0.009466 \| 0.997835 \| 6 \| 54 \| Jasmonic acid mediated signaling pathway \| BP \| 0.009466 \| \| GO:0006857 \| 0.009853 \| 0.998121 \| 5 \| 39 \| Oligopeptide transport \| BP \| 0.009853 \| |
| --- | --- | --- | --- | --- | --- | --- | --- | --- | --- | --- | --- | --- | --- | --- | --- | --- | --- | --- | --- | --- | --- | --- | --- | --- | --- | --- | --- | --- | --- | --- | --- | --- | --- | --- | --- | --- | --- | --- | --- | --- | --- | --- | --- | --- | --- | --- | --- | --- | --- | --- | --- | --- | --- | --- | --- | --- | --- | --- | --- | --- | --- | --- | --- | --- | --- | --- | --- | --- | --- | --- | --- | --- | --- | --- | --- | --- | --- | --- | --- | --- | --- | --- | --- | --- | --- | --- | --- | --- | --- | --- | --- | --- | --- | --- | --- | --- | --- | --- | --- | --- | --- | --- | --- | --- | --- | --- | --- | --- | --- | --- | --- | --- | --- | --- | --- | --- | --- | --- | --- | --- | --- | --- | --- | --- | --- | --- | --- | --- | --- | --- | --- | --- | --- | --- | --- | --- | --- | --- | --- | --- | --- | --- | --- | --- | --- | --- | --- | --- | --- | --- | --- | --- | --- | --- | --- | --- | --- | --- | --- | --- | --- | --- | --- | --- | --- | --- | --- | --- | --- | --- | --- | --- | --- | --- | --- | --- | --- | --- | --- | --- | --- | --- | --- | --- | --- | --- | --- | --- | --- | --- | --- | --- | --- | --- | --- | --- | --- | --- | --- | --- | --- | --- | --- | --- | --- | --- | --- | --- | --- | --- | --- | --- | --- | --- | --- | --- | --- | --- | --- | --- | --- | --- | --- | --- | --- | --- | --- | --- | --- | --- | --- | --- | --- | --- | --- | --- | --- | --- | --- | --- | --- | --- | --- | --- | --- | --- | --- | --- | --- | --- | --- | --- | --- | --- | --- | --- | --- | --- | --- | --- | --- | --- | --- | --- | --- | --- | --- | --- | --- | --- | --- | --- | --- | --- | --- | --- | --- | --- | --- | --- | --- | --- | --- | --- | --- | --- | --- | --- | --- | --- | --- | --- | --- | --- | --- | --- | --- | --- | --- | --- | --- | --- | --- | --- | --- | --- | --- | --- | --- | --- | --- | --- | --- | --- | --- | --- | --- | --- | --- | --- | --- | --- | --- | --- | --- | --- | --- | --- | --- | --- | --- | --- | --- | --- | --- | --- | --- | --- | --- | --- | --- | --- | --- | --- | --- | --- | --- | --- | --- | --- | --- | --- | --- | --- | --- | --- | --- | --- | --- | --- | --- | --- | --- | --- | --- | --- | --- | --- | --- | --- | --- | --- | --- | --- | --- | --- | --- | --- | --- | --- | --- | --- | --- | --- | --- | --- | --- | --- | --- | --- | --- | --- | --- | --- | --- | --- | --- | --- | --- | --- | --- | --- | --- | --- | --- | --- | --- | --- | --- | --- | --- | --- | --- | --- | --- | --- | --- | --- | --- | --- | --- | --- | --- | --- | --- | --- | --- | --- | --- | --- | --- | --- |

**Supplementary Table S3.** **List_C2**

| Category | Overrepresented_p-value | Underrepresented_p-value | NumDEInCat | NumInCat | Term | Ontology | Padj |
| --- | --- | --- | --- | --- | --- | --- | --- |
| GO:0006606 | 6.34E-12 | 1 | 18 | 50 | Protein import into nucleus | BP | 6.34E-12 |
| GO:0009734 | 7.63E-07 | 1 | 13 | 54 | Auxin-activated signaling pathway | BP | 7.63E-07 |
| GO:0006351 | 4.42E-06 | 0.999998 | 55 | 706 | Transcription, DNA-templated | BP | 4.42E-06 |
| GO:0001510 | 5.50E-05 | 0.999992 | 9 | 40 | RNA methylation | BP | 5.50E-05 |
| GO:0010413 | 0.000102 | 0.999982 | 10 | 51 | Glucuronoxylan metabolic process | BP | 0.000102 |
| GO:0009834 | 0.000175 | 0.999984 | 6 | 19 | Plant-type secondary cell wall biogenesis | BP | 0.000175 |
| GO:0006364 | 0.000205 | 0.999936 | 17 | 144 | rRNA processing | BP | 0.000205 |
| GO:0045492 | 0.000215 | 0.999958 | 10 | 56 | Xylan biosynthetic process | BP | 0.000215 |
| GO:0010089 | 0.000361 | 0.999961 | 6 | 22 | Xylem development | BP | 0.000361 |
| GO:0009718 | 0.000389 | 0.999982 | 4 | 10 | Anthocyanin-containing compound biosynthetic process | BP | 0.000389 |
| GO:0009560 | 0.000438 | 0.999896 | 11 | 71 | Embryo sac egg cell differentiation | BP | 0.000438 |
| GO:0006626 | 0.000694 | 0.999873 | 8 | 45 | Protein targeting to mitochondrion | BP | 0.000694 |
| GO:0042026 | 0.000706 | 0.999961 | 4 | 10 | Protein refolding | BP | 0.000706 |
| GO:0007018 | 0.001019 | 0.999753 | 10 | 64 | Microtubule-based movement | BP | 0.001019 |
| GO:0006406 | 0.001126 | 0.99981 | 7 | 34 | mRNA export from nucleus | BP | 0.001126 |
| GO:0010564 | 0.001553 | 0.999826 | 5 | 19 | Regulation of cell cycle process | BP | 0.001553 |
| GO:0009220 | 0.002371 | 0.999404 | 9 | 61 | Pyrimidine ribonucleotide biosynthetic process | BP | 0.002371 |
| GO:0000741 | 0.003615 | 0.999646 | 4 | 14 | Karyogamy | BP | 0.003615 |
| GO:0009926 | 0.004174 | 0.999385 | 5 | 24 | Auxin polar transport | BP | 0.004174 |
| GO:0051301 | 0.004946 | 0.999049 | 6 | 34 | Cell division | BP | 0.004946 |
| GO:0009165 | 0.005054 | 0.999212 | 5 | 26 | Nucleotide biosynthetic process | BP | 0.005054 |
| GO:0008361 | 0.00508 | 0.999428 | 4 | 17 | Regulation of cell size | BP | 0.00508 |
| GO:0010076 | 0.009368 | 0.999741 | 2 | 4 | Maintenance of floral meristem identity | BP | 0.009368 |

**Supplementary Table S4.** **List_C3**

| Category | Overrepresented_p-value | Underrepresented_p-value | NumDEInCat | NumInCat | Term | Ontology | Padj |
| --- | --- | --- | --- | --- | --- | --- | --- |
| GO:0006457 | 0.000517 | 0.999881 | 10 | 175 | Protein folding | BP | 0.000517 |
| GO:0008152 | 0.001622 | 0.999922 | 3 | 15 | Metabolic process | BP | 0.001622 |
| GO:0042254 | 0.004126 | 0.999714 | 3 | 20 | Ribosome biogenesis | BP | 0.004126 |
| GO:0048833 | 0.007951 | 1 | 1 | 1 | Specification of floral organ number | BP | 0.007951 |

**Supplementary Table S5.** **List_C4**

| Category | Overrepresented_p-value | Underrepresented_p-value | NumDEInCat | NumInCat | Term | Ontology | Padj |
| --- | --- | --- | --- | --- | --- | --- | --- |
| GO:0048544 | 7.00E-08 | 1 | 9 | 65 | Recognition of pollen | BP | 7.00E-08 |
| GO:0046246 | 0.008794 | 1 | 1 | 1 | Terpene biosynthetic process | BP | 0.008794 |

**Supplementary Table S6.** **List_C**5

| Category | Overrepresented_p-value | Underrepresented_p-value | NumDEInCat | NumInCat | Term | Ontology | Padj |
| --- | --- | --- | --- | --- | --- | --- | --- |
| GO:0005975 | 5.39E-09 | 1 | 77 | 360 | Carbohydrate metabolic process | BP | 5.39E-09 |
| GO:0006073 | 5.03E-07 | 1 | 16 | 37 | Cellular glucan metabolic process | BP | 5.03E-07 |
| GO:0071555 | 4.98E-06 | 0.999998 | 37 | 154 | Cell wall organization | BP | 4.98E-06 |
| GO:0030245 | 4.06E-05 | 0.999996 | 9 | 18 | Cellulose catabolic process | BP | 4.06E-05 |
| GO:0006952 | 0.000116 | 0.999959 | 23 | 109 | Defense response | BP | 0.000116 |
| GO:0006559 | 0.00015 | 0.999992 | 6 | 9 | L-phenylalanine catabolic process | BP | 0.00015 |
| GO:0009835 | 0.000163 | 0.999991 | 6 | 9 | Fruit ripening | BP | 0.000163 |
| GO:0006629 | 0.000257 | 0.999897 | 27 | 119 | Lipid metabolic process | BP | 0.000257 |
| GO:0009800 | 0.000363 | 0.999985 | 5 | 7 | Cinnamic acid biosynthetic process | BP | 0.000363 |
| GO:0045490 | 0.000992 | 0.999677 | 16 | 61 | Pectin catabolic process | BP | 0.000992 |
| GO:0016998 | 0.001404 | 0.999741 | 8 | 24 | Cell wall macromolecule catabolic process | BP | 0.001404 |
| GO:0006869 | 0.001492 | 0.999551 | 13 | 56 | Lipid transport | BP | 0.001492 |
| GO:0046274 | 0.001544 | 0.999717 | 8 | 22 | Lignin catabolic process | BP | 0.001544 |
| GO:0006308 | 0.001569 | 1 | 3 | 3 | DNA catabolic process | BP | 0.001569 |
| GO:0006032 | 0.001582 | 0.999702 | 8 | 24 | Chitin catabolic process | BP | 0.001582 |
| GO:0010337 | 0.001591 | 1 | 3 | 3 | Regulation of salicylic acid metabolic process | BP | 0.001591 |
| GO:0052542 | 0.001945 | 0.999818 | 5 | 10 | Defense response by callose deposition | BP | 0.001945 |
| GO:0045017 | 0.001972 | 0.999829 | 5 | 9 | Glycerolipid biosynthetic process | BP | 0.001972 |
| GO:0009607 | 0.002199 | 0.999269 | 14 | 63 | Response to biotic stimulus | BP | 0.002199 |
| GO:0006857 | 0.002554 | 0.999292 | 11 | 39 | Oligopeptide transport | BP | 0.002554 |
| GO:0009611 | 0.002813 | 0.999042 | 14 | 61 | Response to wounding | BP | 0.002813 |
| GO:0006979 | 0.004171 | 0.997838 | 30 | 170 | Response to oxidative stress | BP | 0.004171 |
| GO:0006662 | 0.0045 | 0.998729 | 10 | 38 | Glycerol ether metabolic process | BP | 0.0045 |
| GO:0019318 | 0.004922 | 0.999622 | 4 | 7 | Hexose metabolic process | BP | 0.004922 |
| GO:0015976 | 0.005162 | 0.999842 | 3 | 4 | Carbon utilization | BP | 0.005162 |
| GO:0015706 | 0.005616 | 0.998502 | 9 | 32 | Nitrate transport | BP | 0.005616 |
| GO:0048544 | 0.005642 | 0.997836 | 15 | 65 | Recognition of pollen | BP | 0.005642 |
| GO:0010167 | 0.005998 | 0.998556 | 8 | 26 | Response to nitrate | BP | 0.005998 |
| GO:0010363 | 0.00685 | 0.997402 | 14 | 63 | Regulation of plant-type hypersensitive response | BP | 0.00685 |
| GO:0006612 | 0.007258 | 0.997227 | 14 | 63 | Protein targeting to membrane | BP | 0.007258 |
| GO:0002237 | 0.008528 | 0.998062 | 7 | 22 | Response to molecule of bacterial origin | BP | 0.008528 |
| GO:0009693 | 0.009692 | 0.997719 | 7 | 23 | Ethylene biosynthetic process | BP | 0.009692 |

**Supplementary Table S7.** **List_C6**

| Category | Overrepresented_p-value | Underrepresented_p-value | NumDEInCat | NumInCat | Term | Ontology | Padj |
| --- | --- | --- | --- | --- | --- | --- | --- |
| GO:0009765 | 5.06E-08 | 1 | 9 | 33 | Photosynthesis, light harvesting | BP | 5.06E-08 |
| GO:0018298 | 1.42E-06 | 1 | 9 | 49 | Protein-chromophore linkage | BP | 1.42E-06 |
| GO:0010103 | 0.000164 | 0.999975 | 8 | 61 | Stomatal complex morphogenesis | BP | 0.000164 |
| GO:0009415 | 0.000241 | 0.999996 | 3 | 6 | Response to water | BP | 0.000241 |
| GO:0031640 | 0.000356 | 1 | 2 | 2 | Killing of cells of other organism | BP | 0.000356 |
| GO:0031408 | 0.000891 | 0.999906 | 5 | 29 | Oxylipin biosynthetic process | BP | 0.000891 |
| GO:0006979 | 0.001128 | 0.999668 | 12 | 170 | Response to oxidative stress | BP | 0.001128 |
| GO:0016123 | 0.001615 | 0.999987 | 2 | 3 | Xanthophyll biosynthetic process | BP | 0.001615 |
| GO:0015824 | 0.002353 | 0.999877 | 3 | 12 | Proline transport | BP | 0.002353 |
| GO:0009607 | 0.002902 | 0.999477 | 6 | 63 | Response to biotic stimulus | BP | 0.002902 |
| GO:0009081 | 0.004168 | 0.999924 | 2 | 4 | Branched-chain amino acid metabolic process | BP | 0.004168 |
| GO:0048441 | 0.006757 | 0.999818 | 2 | 5 | Petal development | BP | 0.006757 |
