# Supplementary figures and images for: Tomato SlBL4 plays an important role in fruit pedicel organogenesis and abscission
Source: Hortic Res. 2021 Apr 1;8:78. doi: 10.1038/s41438-021-00515-0 (PMC8012377; doi:10.1038/s41438-021-00515-0)

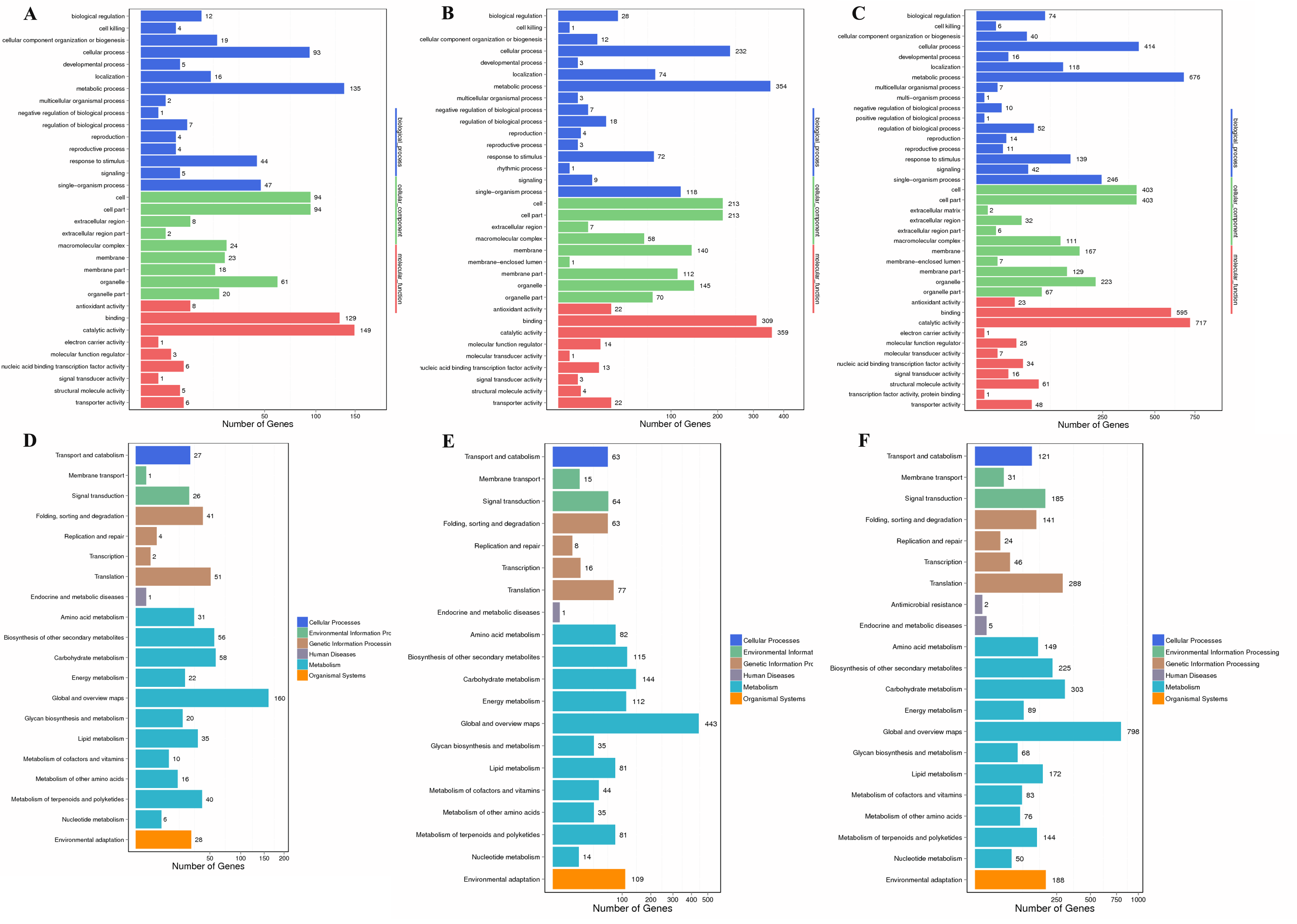

Supplement: Supplementary file 2 — Supplementary figure [file 41438_2021_515_MOESM2_ESM.tif]
